# Efficient uncertainty quantification in a spatially multiscale model of pulmonary arterial and venous hemodynamics

**DOI:** 10.1007/s10237-024-01875-x

**Published:** 2024-07-29

**Authors:** M. J. Colebank, N. C. Chesler

**Affiliations:** Department of Biomedical Engineering, Edwards Lifesciences Foundation Cardiovascular Innovation and Research Center, University of California, Irvine, CA USA

**Keywords:** Uncertainty quantification, Pulse-wave propagation, Hemodynamics, Sensitivity analysis, Multiscale modeling

## Abstract

Pulmonary hypertension (PH) is a debilitating disease that alters the structure and function of both the proximal and distal pulmonary vasculature. This alters pressure-flow relationships in the pulmonary arterial and venous trees, though there is a critical knowledge gap in the relationships between proximal and distal hemodynamics in disease. Multiscale computational models enable simulations in both the proximal and distal vasculature. However, model inputs and measured data are inherently uncertain, requiring a full analysis of the sensitivity and uncertainty of the model. Thus, this study quantifies model sensitivity and output uncertainty in a spatially multiscale, pulse-wave propagation model of pulmonary hemodynamics. The model includes fifteen proximal arteries and twelve proximal veins, connected by a two-sided, structured tree model of the distal vasculature. We use polynomial chaos expansions to expedite sensitivity and uncertainty quantification analyses and provide results for both the proximal and distal vasculature. We quantify uncertainty in blood pressure, blood flow rate, wave intensity, wall shear stress, and cyclic stretch. The latter two are important stimuli for endothelial cell mechanotransduction. We conclude that, while nearly all the parameters in our system have some influence on model predictions, the parameters describing the density of the microvascular beds have the largest effects on all simulated quantities in both the proximal and distal arterial and venous circulations.

**Supplementary Information:**

The online version contains supplementary material available at 10.1007/s10237-024-01875-x.

## Introduction

The pulmonary circulation supports the same cardiac output as the systemic circulation but with substantially lower pressure magnitudes (between 5 and 20 mmHg) (Gu et al. 2023). Elevated pulmonary blood pressures constitute pulmonary hypertension (PH), a debilitating, often fatal disease that is attributed to vascular remodeling and causes right ventricle (RV) dysfunction if left unmanaged. The disease is defined by a resting mean pulmonary arterial blood pressure $\ge$ 20 mmHg measured by right heart catheterization and is a comorbidity in 36–83% of all adults with left-sided heart failure (Allen et al. 2023). While PH secondary to left-sided heart failure (World Health Organization (WHO) group II PH) is prevalent, there is still an unmet need in understanding the hemodynamic drivers and consequences of group II PH (Allen et al. 2023).

A majority of PH research has focused on the proximal and distal pulmonary arteries, as RV dysfunction can be correlated with elevated proximal arterial pressures (Vonk Noordegraaf et al. 2017). For example, pulmonary arterial hypertension (PAH) severity is linked to increased distal pulmonary arterial wall thickness, lower proximal arterial compliance, and eventual hemodynamic un-coupling of the RV and proximal arteries (Bellofiore and Chesler 2013). These measures of dysfunction are also correlated with dysfunctional vascular mechanotransduction, which involves the translation of hemodynamics into cell signaling cascades. In contrast, less is known about the role of the pulmonary microvasculature surrounding the alveoli in the lung, which is hypothesized to remodel in PH due to lung disease (Gu et al. 2023). Severe cases of heart failure can also result in capillary remodeling, distal arterial stiffening, and RV deterioration (Guazzi et al. 2020; Allen et al. 2023). The exact mechanism of this transition in unknown, but is likely attributed to changes in mechanical forces, such as wall shear stress (WSS) and cyclic stretch (CS), which cause malicious changes in mechanotransduction cascades (Allen et al. 2023). These various forms of PH are heterogeneous and cause both cardiac and vascular dysfunction at multiple spatial scales. Disease diagnosis and prognosis rely on multiple data modalities (e.g., catheterization, imaging, echocardiography) that cannot be integrated easily.

Computational fluid dynamics models have provided significant insight into systemic hemodynamics by integrating multimodal clinical data (Olufsen 1999; Huberts et al. 2014; Mynard and Smolich 2015; Eck et al. 2017). These models have also been applied to the pulmonary circuit, including fully explicit three-dimensional (3D) (Bordones et al. 2018; Yang et al. 2019) and reduced order (Qureshi et al. 2014; Clark and Tawhai 2018; Colebank et al. 2021; Bartolo et al. 2022) hemodynamics models. These mechanistic models can be solved in subject-specific geometries from imaging data and have potential as a noninvasive tool for disease monitoring (Corral-Acero et al. 2020; Morrison et al. 2023). In particular, one-dimensional (1D) hemodynamic models provide network-level insight into pressure-flow dynamics (Qureshi et al. 2014; Bartolo et al. 2022), as well as simulations of wave travel and wave reflections, which correlate with PH severity (Quail et al. 2015; Qureshi and Hill 2015; Su et al. 2016). Several simulation studies focusing on the pulmonary circulation have quantified spatial multiscale phenomenon, including distal arterial (Colebank et al. 2021) and venous (Qureshi et al. 2014; Clark and Tawhai 2018; Bartolo et al. 2022) hemodynamics. However, few studies have quantified the uncertainties in these models (Huberts et al. 2014; Eck et al. 2016; Brault et al. 2017), and, to the authors’ knowledge, none have investigated the sensitivity and uncertainty of a multiscale hemodynamics model. These latter analyses are imperative, as modeling and simulation undergo heavy scrutiny before advancing to medical device or clinical applications (Erdemir et al. 2020; Morrison et al. 2023).

We address this gap in the field by conducting a formal sensitivity and uncertainty analysis on a spatially multiscale, two-sided model of the pulmonary circulation. We use the 1D hemodynamics model developed by Qureshi et al. (2014) and recently innovated on by Bartolo et al. (2022) to study group II PH. The model simulates nonlinear pulmonary arterial and venous hemodynamics in the proximal vasculature (i.e., the first 2–3 generations of arteries and veins) and uses the structured tree model to generate an artificial, self-similar bifurcating tree representative of the distal vasculature (Qureshi et al. 2014; Bartolo et al. 2022). We employ polynomial chaos expansions (PCEs) to circumvent high computational cost and provide Sobol’ indices to measure parameter influence on pressure, flow rate, WSS, and CS in both the proximal and distal arteries and veins. We subsequently provide insight into the uncertainties in wave propagation in the arterial and venous systems. Our analysis identifies the biophysical parameters of the model that are most influential on proximal and distal arterial and venous hemodynamics. Importantly, our results suggest that microvascular structure (e.g., the number of arterioles and venules) is paramount to both proximal and distal vascular function. We provide uncertainty bounds for hemodynamic and biomechanical stimuli from the model at different spatial scales, presenting new details for future studies that seek to calibrate this model to patient data. Lastly, our findings support experimental conditions for *in vitro* studies that require appropriate stimuli to interrogate pulmonary vascular mechanotransduction.

## Materials and methods

### Vascular geometry

**Fig. 1:**
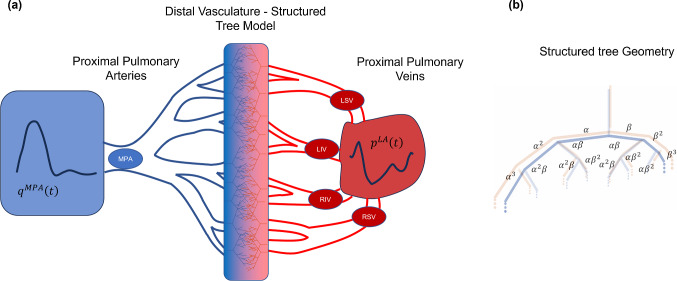
Schematic of computational model geometry. **a** A pulmonary artery inflow profile is provided as a boundary condition to the MPA and drives flow through the fifteen proximal arteries. A left atrial pressure waveform is provided as a pressure boundary condition for four proximal pulmonary veins, which are connected to an additional generation of veins. The proximal arteries and veins are connected by the structured tree model, which includes the distal vasculature. **b** A pictorial representation of the structured tree model and how the parameters $\alpha$ and $\beta$ are used to determine vessel radii. Note that there are both arterial and venous structured trees, which have the same geometry. MPA: main pulmonary artery; LSV: left superior vein; LIV: left inferior vein; RSV: right superior vein; RIV: right inferior vein

The model operates on two vascular domains, as shown in Fig. 1. The first domain includes pulmonary arteries (n = 15) up to the segmental level, as well as the first two generations of pulmonary veins (n = 12). This constitutes the *proximal* vasculature where the nonlinear 1D hemodynamic equations are solved. Each arterial and venous vessel includes a radius and length, as documented in Table 1, based on the findings in Mynard and Smolich (2015). The arteries and veins at the end of the proximal vasculature are deemed *terminal branches* herein. The axial domain for each vessel is $0\le x\le L$, with $L$ (cm) being the length of the vessel.

**Table 1:** Vessel network used in this work based on Mynard and Smolich (2015)

Branch name	Length (cm)	Radius (cm)	Parent: Daughters
Arteries			
Main Pulmonary Artery (MPA)	4.30	1.350	None: LPA, RPA
Left Pulmonary Artery (LPA)	2.50	0.900	MPA: LIA, LSA
Right Pulmonary Artery (RPA)	5.75	1.100	MPA: RIA, RSA
Left Inferior Pulmonary Artery (LIA)	2.15	0.842	LPA: LIA D1, LIA D2
Left Superior Pulmonary Artery (LSA)	1.23	0.481	LPA: LSA D1, LSA D2
Right Inferior Pulmonary Artery (RIA)	2.35	0.922	RPA: RIA D1, RIA D2
Right Superior Pulmonary Artery (RSA)	1.92	0.755	RPA: RSA D1, RSA D2
LIA Daughter 1 (LIA D1)	1.93	0.757	LIA: LIV D1
LIA Daughter 2 (LIA D2)	1.31	0.514	LIA: LIV D2
LSA Daughter 1 (LSA D1)	1.10	0.433	LSA: LSV D1
LSA Daughter 2 (LSA D2)	0.75	0.293	LSA: LSV D2
RIA Daughter 1 (RIA D1)	2.11	0.829	RIA: RIV D1
RIA Daughter 2 (RIA D2)	1.43	0.562	RIA: RIV D2
RSA Daughter 1 (RSA D1)	1.17	0.460	RSA: RSV D1
RSA Daughter 2 (RSA D2)	1.55	0.610	RSA: RSV D2
Veins			
Left Inferior Pulmonary Vein (LIV)	2.15	0.641	None: LIV D1, LIV D2
Left Superior Pulmonary Vein (LSV)	1.23	0.716	None: LSV D1, LSV D2
Right Inferior Pulmonary Vein (RIV)	2.35	0.864	None: RIV D1, RIV D2
Right Superior Pulmonary Vein (RSV)	1.92	0.824	None: RSV D1, RSV D2
LIV Daughter 1 (LIV D1)	1.93	0.576	LIV: LIA D1
LIV Daughter 2 (LIV D2)	1.31	0.391	LIV: LIA D2
LSV Daughter 1 (LSV D1)	1.10	0.643	LSV: LSA D1
LSV Daughter 2 (LSV D2)	0.75	0.436	LSV: LSA D2
RIV Daughter 1 (RIV D1)	2.11	0.777	RIV: RIA D1
RIV Daughter 2 (RIV D2)	1.43	0.527	RIV: RIA D2
RSV Daughter 1 (RSV D1)	1.73	0.740	RSV: RSA D1
RSV Daughter 2 (RSV D2)	1.17	0.502	RSV: RSA D2

The *distal* vasculature is constructed using the structured tree model (Olufsen 1999; Qureshi et al. 2014; Bartolo et al. 2022). Although the original formulation of the structured tree was based on the systemic vasculature (Olufsen 1999), numerous studies have also identified a self-similar, scalable structure within the pulmonary vasculature (Rahaghi et al. 2016; Chambers et al. 2020). The arterial and venous beds are assumed to follow a self-similar, bifurcating structure, parameterized by five geometric parameters: $\alpha$ and $\beta$ (dimensionless), the major and minor radii scaling factors in the structured tree; ${{\ell}}_{rr}^{A}$ and ${{\ell}}_{rr}^{V}$ (dimensionless), the length-to-radius ratios of the arterial and venous trees; and ${r}_{min}$ (cm), the minimum radius cutoff for where the arterial and venous beds meet. Each vessel in the structured tree is described by

1 $$r_{ij} = \alpha^{i} \beta^{j} r_{{{\text{term}}}} , \ \ \ L_{ij}^{k} = r_{ij} \ell_{rr}^{k} , \ \ \ k = A,V$$

where ${r}_{ij}$ and ${L}_{ij}^{k}$ are the radius (cm) and length (cm) in the arterial ($k=A$) or venous ($k=V$) bed. Details regarding the self-similarity principles can be found in the original work by Olufsen (1999).

### Proximal vessel fluid mechanics

We simulate proximal pulmonary hemodynamics using a 1D model of the large arteries and veins, as developed by Qureshi et al. (2014) and Bartolo et al. (2022). In short, we assume that the blood is Newtonian and homogenous, and that flow is predominantly inertial, axially dominant, laminar, and axisymmetric, with no-swirl, resulting in only axial $x$ (cm) and temporal $t$ (s) dynamics. Each blood vessel is assumed to be cylindrical and impermeable with a circular cross section. The resulting mass conservation and momentum balance equations are

2 $$\frac{\partial A}{{\partial t}} + \frac{\partial q}{{\partial x}} = 0$$

and

3 $$\frac{\partial q}{{\partial t}} + \frac{{\left( {\gamma + 2} \right)}}{{\left( {\gamma + 1} \right)}}\frac{\partial }{\partial x}\left( {\frac{{q^{2} }}{A}} \right) + \frac{A}{\rho }\frac{\partial p}{{\partial x}} = - 2\pi \nu \left( {\gamma + 2} \right)\frac{q}{A}$$

where $A\left(x,t\right)$ (cm^2^) is the dynamic vessel area, $q\left(x,t\right)$ (cm^3^/s) is the flow rate, and $p\left(x,t\right)$ (g cm/s^2^) is the transmural pressure. The blood density and kinematic viscosity are assumed constant in the large vessels, with $\rho =1.055$ (g/cm^3^) and $\nu =3.03\times {10}^{-2}$ (cm^2^/s), respectively. We assume a power-law velocity profile with $\gamma =9$, providing a blunt velocity profile in the center of each vessel that decreases to zero to satisfy the no-slip condition at the vessel wall (van de Vosse and Stergiopulos 2011). For the proximal wall mechanics, we assume that vessels are thin-walled, homogenous, and orthotropic, and follow a linearly elastic stress–strain relationship (Bartolo et al. 2022). This is modeled by

4 $$p\left( {x,t} \right) = \frac{4}{3}\left( {\frac{Eh}{{r_{0} }}} \right)\left( {\sqrt {A/A_{0} } - 1} \right)$$

where ${A}_{0}=\pi {r}_{0}^{2}$ is the reference area (cm^2^), $E$ (g cm^2^/s) is the Young’s modulus in the circumferential direction, and $h$ (cm) is the wall thickness. We assume that the proximal arteries have the same, constant material properties, ${K}_{A}={E}_{A}{h}_{A}/{r}_{0,A}$ (g cm^2^/s), while all the proximal veins have their own constant, venous-specific material properties, ${K}_{V}={E}_{V}{h}_{V}/{r}_{0,V}$ (g cm^2^/s) (Qureshi et al. 2014). The proximal vessel equations are discretized and solved using the two-step Lax-Wendroff scheme (Olufsen 1999). Numerical simulations are run through a combination of FORTRAN90 and C +  + using a MATLAB (Natick, MA) wrapper file. It should be noted that pressure is calculated in CGS units and then converted to mmHg (1 mmHg = 1333.22 g cm^2^/s) to make results clearer. We use a discretization of $\Delta x=0.125$ (cm) and $\Delta t\approx 1.04\times {10}^{-4}$ (s) which provides numerically accurate solutions across the parameter domain while still satisfying the Courant-Friedrich-Lewy (CFL) condition (Olufsen 1999). The model is stopped once beat-to-beat convergence (pressure error $\le$ 1 g cm^2^/s) is reached, and the last cycle of the solution is used for all analyses.

### Distal vessel fluid mechanics

Whereas the proximal vascular fluid mechanics include both inertial and viscous forces, hemodynamics in the distal vasculature are assumed to be viscous dominant. We assume that pressure and flow in the structured tree branches are periodic with each heartbeat and subsequently use the frequency domain representation of pressure, $P\left(x,{\omega }_{k}\right)$, and flow rate, $Q\left(x,{\omega }_{k}\right)$, respectively, for each frequency ${\omega }_{k}=2\pi k/T$ (rad/s). This leads to a *linear* mass conservation and momentum balance system given by the expressions

5 $$i\omega_{k} CP\left( {x,\omega_{k} } \right) + \frac{{\partial Q\left( {x,\omega_{k} } \right)}}{\partial x} = 0,  \ \ \ C = \frac{3}{2}\frac{{\left( {\pi r_{0,ij}^{2} } \right)}}{{K_{ST} }}$$

and

6 $$\begin{aligned} & i\omega_{k} Q\left( {x,\omega_{k} } \right) + \frac{{\left( {\pi r_{0,ij}^{2} } \right)}}{\rho }\left( {1 - \frac{{2J_{1} \left( {w_{0} } \right)}}{{w_{0} J_{0} \left( {w_{0} } \right)}}} \right)\frac{{\partial P\left( {x,\omega_{k} } \right)}}{\partial x} = 0, \\ & \ w_{0} = i^{3} r_{0,ij}^{2} \omega_{k} /\mu^{{{\text{ST}}}} \end{aligned}$$

The above equations depend on ${K}_{ST}=Eh/{r}_{0,ij}$ (g cm^2^/s), the material properties of the vascular wall for both the arterial and venous structured trees, the imaginary unit $i=\sqrt{-1}$, and the first- and zeroth-order Bessel functions, ${J}_{1}$ and ${J}_{0}$, respectively. The structured tree viscosity, ${\mu }^{ST}=\mu \left({r}_{0,ij}\right)$, is radius dependent, as described previously (Pries et al. 1992; Bartolo et al. 2022), where ${r}_{0,ij}$ is the radius value for the $ij$-th branch of the structured tree. The compliance, $C$, is derived using the same linear relationship introduced in Eq. (3) under the assumption that $Eh\gg p{r}_{0}$ (Olufsen 1999). Equation (5) can be differentiated with respect to $x$ and used in Eq. (6) to give a system of wave equations in $P\left(x,{\omega }_{k}\right)$ and $Q(x,{\omega }_{k})$. Their solution can be computed analytically in terms of sine and cosine functions, as described elsewhere (Qureshi et al. 2014; Bartolo et al. 2022).

The numerical solutions for $P\left(x,{\omega }_{k}\right)$ and $Q\left(x,{\omega }_{k}\right)$ require a pressure-flow relationship. As originally discussed by (Qureshi et al. 2014), the arterial and venous structured trees are linked using admittance, which is the inverse of impedance and generally expressed as $Y=Q/P$. Using the analytical solutions for hemodynamics and the structured tree geometry, the pressure and flow rate at the inlet ($x=0$) and outlet $(x=L$) of any vessel can be determined by the admittance relationship

7 $$\left[ {\begin{array}{*{20}c} {Q\left( {0,\omega_{k} } \right)} \\ \\ {Q\left( {L,\omega_{k} } \right)} \\ \end{array} } \right] = {\varvec{Y}}\left( {\omega_{k} } \right)\left[ {\begin{array}{*{20}c} {P\left( {0,\omega_{k} } \right)} \\ \\ {P\left( {L,\omega_{k} } \right)} \\ \end{array} } \right]$$

where ${\varvec{Y}}\left({\omega }_{k}\right)$ is the 2 × 2 admittance matrix

8 $${\varvec{Y}}\left( {\omega_{k} } \right) = \frac{{ig_{{\omega_{k} }} }}{{\sin \left( {\omega_{k} L/c} \right)}}\left[ {\begin{array}{*{20}c} { - \cos \left( {\omega_{k} L/c} \right)} & 1 \\ 1 & { - \cos \left( {\omega_{k} L/c} \right)} \\ \end{array} } \right],$$

9 $$g_{{\omega_{k} }} = \sqrt {\frac{{C\left( {\pi r_{0,ij}^{2} } \right)}}{\rho }\left( {1 - \frac{{2J_{1} \left( {w_{0} } \right)}}{{w_{0} J_{0} \left( {w_{0} } \right)}}} \right)}$$

Note that at ${\omega }_{k}=0$, we obtain a Poiseuille-like admittance matrix

10 $${\varvec{Y}}\left( {\omega_{k} = 0} \right) = \frac{{\pi r_{0,ij}^{4} }}{{8\mu^{{{\text{ST}}}} L_{ij} }}\left[ {\begin{array}{*{20}c} 1 & { - 1} \\ { - 1} & 1 \\ \end{array} } \right]$$

where ${{r}_{0}}_{ij}$ and ${L}_{ij}$ denote the reference radius and length of the vessel in the structured tree and ${\mu }^{\text{ST}}$ is the radius dependent viscosity (Bartolo et al. 2022). The admittance throughout the structured tree is dependent on the structured tree parameters $\theta^{{{\text{ST}}}} = \left\{ {K_{{{\text{ST}}}} ,\alpha ,\beta ,\ell_{rr}^{A} , \ell_{rr}^{V} ,r_{{{\text{min}}}} } \right\}$.

### Multiscale coupling

The proximal arteries and veins are coupled to the distal structured tree beds using the “grand admittance” of the structured tree (Bartolo et al. 2022). To link the two models, the grand admittance matrix is used as a frequency-domain boundary condition to the proximal arteries and veins via a convolution integral. The proximal artery pressure and flow rate on the arterial and venous sides are calculated (respectively) using the relationship

11 $$q_{A} \left( {L,t} \right) = \mathop \int \limits_{0}^{T} \left( {y_{11} \left( t \right)p_{A} \left( {L,t - \tau } \right) + y_{12} \left( t \right)p_{V} \left( {0,t - \tau } \right)} \right)d\tau$$

12 $$q_{V} \left( {0,t} \right) = \mathop \int \limits_{0}^{T} \left( {y_{21} \left( t \right)p_{A} \left( {L,t - \tau } \right) + y_{22} \left( t \right)p_{V} \left( {0,t - \tau } \right)} \right)d\tau$$

The above expressions depend on the components of the admittance matrix, ${y}_{ij}(t)$, which are the inverse Fourier transformed version of ${Y}_{ij}\left({\omega }_{k}\right)$.

Once the large artery equations have been solved, the frequency domain variables $P\left(x,{\omega }_{k}\right)$, $Q\left(x,{\omega }_{k}\right)$, and other hemodynamic quantities derived from these, can be calculated in the structured tree. The Fourier transformed pressure solutions at the connecting terminal proximal arteries, ${P}_{root}^{A}\left({\omega }_{k}\right),$ and veins, ${P}_{root}^{V}\left({\omega }_{k}\right),$ are used to in Eq. (7) to obtain the arterial and venous flow rates at the root of the structured trees. From there, the pressure and flow rate solutions at $x=L$ are computed as

13 $$P\left( {L,\omega_{k} } \right) = \frac{1}{{Y_{12} \left( {\omega_{k} } \right)}}\left( {Q\left( {0,\omega_{k} } \right) - Y_{11} P\left( {0,\omega_{k} } \right)} \right)$$

14 $$Q\left( {L,\omega_{k} } \right) = Y_{21} P\left( {0,\omega_{k} } \right) - Y_{22} P\left( {L,\omega_{k} } \right)$$

Distal vessel hemodynamics are calculated down the $\alpha$-sides and $\beta$-sides of each arterial and venous tree. This reflects the largest and smallest pathways in the structured tree, respectively; i.e., the $\alpha$-side will include the *greatest* number of branches, while the $\beta$-side will include the *fewest* number of branches (Bartolo et al. 2022).

### Inlet and Outlet Boundary Conditions

The mass conservation and momentum balance Eqs. (2–3) constitute a coupled hyperbolic partial differential equation (PDE) system. We require boundary conditions at each proximal vessel inlet ($x=0$) and outlet ($x=L$). At the inlet of the main pulmonary artery (MPA, the first vessel in the network), we enforce a period flow rate boundary condition, ${q}^{\text{MPA}}\left(t\right)$, using magnetic resonance imaging data obtained from the SimVascular webpage^1^ (Colebank et al. 2021). At the proximal vessel junctions, we assume a conservation of flow and a continuity of total pressure

15 $$\begin{aligned} & q_{p} \left( {L,t} \right) = q_{{d_{1} }} \left( {0,t} \right) + q_{{d_{2} }} \left( {0,t} \right)\, \ \ {\text{and}}\, \\  &p_{p} \left( {L,t} \right) = p_{{d_{1} }} \left( {0,t} \right) = p_{{d_{2} }} \left( {0,t} \right) \end{aligned}$$

where the subscripts $p$, ${d}_{1}$, and ${d}_{2}$ denote the parent and child branches, respectively. As mentioned above, the proximal arterial and venous branches are linked together using the grand admittance matrix from the structured tree and the convolution interval defined in Eqs. (11) and (12). Lastly, we prescribe a simulated left-atrial pressure waveform, ${p}^{\text{LA}}\left(t\right)$, at the distal end of each of the four terminal pulmonary veins: the left and right superior pulmonary veins (LSV, RSV) and the left and right inferior pulmonary veins (LIV, RIV). The left-atrial pressure waveform is extracted from a previously published lumped parameter model of the circulation (Colunga et al. 2023).

### Global sensitivity analysis

We use variance-based sensitivity analysis to investigate parameter effects on different model outputs. Let ${\varvec{Z}}=\mathcal{M}\left({\varvec{\theta}}\right)$, represent a quantity of interest from the model $\mathcal{M}$ which depends on the parameters ${\varvec{\theta}}$. Throughout, we assume that the parameters can be mapped to a uniformly distributed random variable on the interval $\left[\text{0,1}\right].$ Under the assumption of $N$ independent input parameters, the model response can be decomposed as

16 $${\mathcal{M}}\left( {\varvec{\theta}} \right) \approx f_{0} + \mathop \sum \limits_{i = 1}^{N} f_{i} \left( {\theta_{i} } \right) + \mathop \sum \limits_{i = 1}^{N} \mathop \sum \limits_{j = i + 1}^{N} f_{ij} \left( {\theta_{i} ,\theta_{j} } \right) + \ldots ,$$

where

17 $$f_{0} = \mathop \int \limits_{0}^{1} {\mathcal{M}}\left( \theta \right)d\theta = {\text{E}}\left[ {\varvec{Z}} \right]$$

18 $$f_{i} = {\text{E}}\left[ {{\varvec{Z}}{|}\theta_{i} } \right] - f_{0}$$

19 $$f_{ij} = {\text{E}}\left[ {{\varvec{Z}}|\theta_{i} ,\theta_{j} } \right] - f_{i} - f_{j} - f_{0}$$

and so on. The notation $\text{E}\left[{\varvec{Z}}|{\theta }_{i}\right]$ represents the expectation of the output conditioned on a known, fixed value of the parameter, ${\theta }_{i}$. The term ${f}_{0}$ represents the average response, the term ${f}_{i}$ is the response attributed to only parameter ${\theta }_{i}$, and the term ${f}_{ij}$ is the response associated with the interaction between ${\theta }_{i}$ and ${\theta }_{j}$. In addition, each ${f}_{i},{f}_{ij},\dots$ term above is constrained to have an expected value of zero, which implies that each decomposition is orthogonal to each other (Eck et al. 2016). We can then write the total variance of the system as

20 $$D\left( {\varvec{Z}} \right) = {\text{Var}}\left[ {\varvec{Z}} \right] = \mathop \int \limits_{0}^{1} \left( {{\mathcal{M}}\left( \theta \right)} \right)^{2} d\theta - f_{0}^{2} .$$

The partial variances, ${D}_{i}\left({\varvec{Z}}\right)$ and ${D}_{ij}\left({\varvec{Z}}\right)$, are then

21 $$D_{i} \left( {\varvec{Z}} \right) = \mathop \int \limits_{0}^{1} f_{i}^{2} \left( {\theta_{i} } \right)d\theta_{i} , \ \ \ D_{ij} \left( {\varvec{Z}} \right) = \mathop \int \limits_{0}^{1} \mathop \int \limits_{0}^{1} f_{ij}^{2} \left( {\theta_{i} ,\theta_{j} } \right)d\theta_{i} d\theta_{j} .$$

Using these definitions, the *first-order* Sobol’ index, ${S}_{i}$, for the parameter ${\theta }_{i}$ is defined as

22 $$S_{i} = \frac{{D_{i} }}{D} = \frac{{{\text{Var}}\left[ {{\text{E}}\left[ {{\varvec{Z}}|\theta_{i} } \right]} \right]}}{{{\text{Var}}\left[ {\varvec{Z}} \right]}}$$

which represents the variance attributed to the parameter ${\theta }_{i}$ alone. The *second-order* and *total-order* Sobol’ indices, ${S}_{ij}$ and ${{S}_{T}}_{i}$, are defined in a similar fashion

23 $$S_{ij} = \frac{{D_{ij} }}{D} = \frac{{{\text{Var}}\left[ {{\text{E}}\left[ {{\varvec{Z}}|\theta_{i} ,\theta_{j} } \right]} \right]}}{{{\text{Var}}\left[ {\varvec{Z}} \right]}}, \,\,\,S_{{T_{i} }} = 1 - \frac{{{\text{Var}}\left[ {{\text{E}}\left[ {{\varvec{Z}}|\theta_{\sim i} } \right]} \right]}}{{{\text{Var}}\left[ {\varvec{Z}} \right]}}$$

where the notation $\text{E}\left[{\varvec{Z}}|{\theta }_{\sim i}\right]$ represents the expected value of the response when all parameters except ${\theta }_{i}$ are allowed to vary. The second-order index, ${S}_{ij}$, accounts for the pairwise interactions that contribute to the variance of the system. The total index, ${{S}_{T}}_{i}$, is the sum of all the partial variances attributed to the parameter ${\theta }_{i}$, including first-order, second-order, and higher-order Sobol’ indices.

### Polynomial chaos expansions

Variance-based sensitivity indices require numerous parameter samples and model evaluations to achieve accurate metrics. For lower-fidelity models, this is feasible; however, the expensive computation time of running a spatially multiscale PDE, such as the one here, limits the number of evaluations feasible. To circumvent this, we use PCEs to speed up the calculation of output uncertainty and Sobol’ indices.

Briefly, the PCE of a model $M\left({\varvec{\theta}}\right)$ can be approximated by the finite truncation

24 $$M\left( {\varvec{\theta}} \right) \approx \mathop \sum \limits_{j = 0}^{{{\mathcal{J}} - 1}} c_{j} {\Psi }_{j} \left( {\varvec{\theta}} \right), \quad {\Psi }_{j} \left( {\varvec{\theta}} \right) = \mathop \prod \limits_{i = 1}^{{\mathcal{K}}} \psi_{i} \left( {\theta_{i} } \right),$$

where ${c}_{j}$ are the polynomial coefficients, ${\Psi }_{j}\left({\varvec{\theta}}\right)$ are the multivariate polynomials defined by the product of multiple, single-variate polynomials ${\psi }_{i}\left({\theta }_{i}\right)$, and $\mathcal{J}=\left(\genfrac{}{}{0pt}{}{n+\mathcal{K}}{n}\right)$ is the number of polynomial basis functions, with $n$ being the number of parameters in the system and $\mathcal{K}$ denoting the polynomial order. The polynomials are chosen to be orthogonal in their prior space, i.e.

25 $$\int \psi_{i} \left( {\theta_{i} } \right)\psi_{j} \left( {\theta_{i} } \right)d\theta_{i} = \left\{ { \begin{array}{*{20}c} {0, i \ne j} \\ {\gamma_{i} , i = j} \\ \end{array} } \right.$$

where the term ${\gamma }_{i}=\text{E}\left[{\psi }_{i}^{2}\right]$ is the normalization factor for the given polynomial family (Eck et al. 2016). The polynomial type is selected based on the prior probability distribution for the parameters. We assume that parameters are uniformly distributed on a scaled $\left[-\text{1,1}\right]$ interval, and subsequently use Legendre polynomials (Eck et al. 2016).

In this study, the quantities of interest are time dependent. We subsequently have a unique set of polynomial coefficients, ${c}_{ij}$*,* that makeup the larger coefficient matrix$,{\varvec{C}}$, which is a $\mathcal{J}\times {n}_{t}$ matrix, where ${n}_{t}$ is the number of time points. Hence, the polynomial matrix, ${\varvec{\Psi}}$ is $1\times \mathcal{J}$, for a single realization of the model. The coefficients for each polynomial at each time point can be determined using either projection or regression techniques (Eck et al. 2016). Here, we employ the regression approach by computing the coefficients using ordinary least squares. Using a set of training data, ${{\varvec{Z}}}^{{\varvec{i}}}=M\left({{\varvec{\theta}}}^{i}\right)$, we can solve the minimization problem for the matrix of polynomial coefficients

26 $${\varvec{J}} = \mathop {{\text{argmin}}}\limits_{{\varvec{C}}} \mathop \sum \limits_{i = 1}^{N} \left( {{\varvec{Z}}^{i} - {{\varvec{\Psi}}}{\varvec{C}}} \right)^{2} ,$$

which gives rise to the vector matrix solution

27 $${\varvec{C}} = \left( {{\tilde{\mathbf{\Psi }}}^{{\mathbf{ \top }}} \user2{ }{\tilde{\mathbf{\Psi }}}\user2{ }} \right)^{ - 1} {\tilde{\mathbf{\Psi }}}^{{\mathbf{ \top }}} {\varvec{Z}},$$

where $\widetilde{{\varvec{\Psi}}}$ is a repeated matrix of polynomials to match each data instance in ${\varvec{Z}}$. Once the coefficients have been determined, the mean of the output, $\text{E}\left[{\varvec{Z}}\right]$, and the variance, $\text{Var}\left[{\varvec{Z}}\right]$, can be calculated as

28 $${\text{E}}\left[ {\varvec{Z}} \right] = {\varvec{c}}_{0} \left( t \right),{\text{ Var}}\left[ {\varvec{Z}} \right] = \mathop \sum \limits_{j = 1}^{{{\mathcal{J}} - 1}} {\varvec{c}}_{j}^{2} \left( t \right)\gamma_{j}$$

where ${{\varvec{c}}}_{0}\left(t\right)$ and ${{\varvec{c}}}_{{\varvec{j}}}\left(t\right)$ are the coefficients of the PCE over the time interval. The Sobol’ indices can be defined in terms of the polynomial coefficients and the polynomial normalization factors. Let ${\mathcal{A}}_{i}$ denote the set of all polynomial coefficients that only depend on ${\theta }_{i}$ (i.e., without any interactions with other parameters up to the polynomial order $\mathcal{K}$), ${\mathcal{A}}_{ij}$ denote the set of all polynomial coefficients that depend on ${\theta }_{i}$ and ${\theta }_{j}$, and let ${{\mathcal{A}}_{T}}_{i}$ denote the set of all polynomials that have any dependence on ${\theta }_{i}$. The first-order, second-order, and total-order Sobol’ indices are then defined as

29 $$S_{i} \left( t \right) = \left[ {\mathop \sum \limits_{{j \in {\mathcal{A}}_{i} }} {\varvec{c}}_{j}^{2} \left( t \right)\gamma_{j} } \right]/{\text{Var}}\left[ {\varvec{Z}} \right] , \quad S_{ij} = \left[ {\mathop \sum \limits_{{j \in {\mathcal{A}}_{ij} }} {\varvec{c}}_{j}^{2} \left( t \right)\gamma_{j} } \right]/{\text{Var}}\left[ {\varvec{Z}} \right],\quad S_{Ti} = \left[ {\mathop \sum \limits_{{j \in {\mathcal{A}}_{Ti} }} {\varvec{c}}_{j}^{2} \left( t \right)\gamma_{j} } \right]/{\text{Var}}\left[ {\varvec{Z}} \right]$$

Since  ${\varvec{Z}}, {S}_{i}$, ${S}_{ij}$, and ${{S}_{T}}_{i}$ are time-dependent, we use the generalized Sobol’ sensitivities (Alexanderian et al. 2020) to calculate parameter effects. The first-, second-, and total-order generalized indices (${\text{GS}}_{i} ,{\text{GS}}_{ij}$ and ${\text{GS}}_{Ti}$, respectively) are

30 $${\text{GS}}_{i} \left( {t_{j} } \right) = \frac{{\int _{0}^{{t_{j} }} S_{i} \left( {\text{t}} \right){\text{Var}}\left[ {\varvec{Z}} \right]{\text{d}}t}}{{\int_{0}^{{t_{j} }} {\text{Var}}\left[ {\varvec{Z}} \right]{\text{d}}t}}, \quad {\text{GS}}_{ij} \left( {t_{j} } \right) = \frac{{\int_{0}^{{t_{j} }} S_{ij} \left( {\text{t}} \right){\text{Var}}\left[ {\varvec{Z}} \right]{\text{d}}t}}{{\int _{0}^{{t_{j} }} {\text{Var}}\left[ {\varvec{Z}} \right]{\text{d}}t}}, \quad {\text{GS}}_{Ti} \left( {t_{j} } \right) = \frac{{\int_{0}^{{t_{j} }} S_{{T_{i} }} \left( {\text{t}} \right){\text{Var}}\left[ {\varvec{Z}} \right]{\text{d}}t}}{{\int_{0}^{{t_{j} }} {\text{Var}}\left[ {\varvec{Z}} \right]{\text{d}}t}}$$

which calculates the Sobol’ indices at $t_{j}$ using information from all previous time points. The value of ${\text{GS}}_{i}, {\text{GS}}_{ij}$ and ${\text{GS}}_{Ti}$ at the final time point $t_{j} = T$, where $T$(s) is the cardiac cycle length, is used as a measure of parameter importance.

### Quantities of interest

We quantify parameter influence and the output uncertainty for several quantities of interest using PCEs. In the proximal vasculature, we consider time-series arterial pressure and arterial flow rate, as well as the proximal wall shear stress (WSS), defined by

31 $${\text{WSS}}_{{{\text{prox}}}} = - \mu \left( {\frac{\partial u}{{\partial r}}} \right)_{r = R} = \mu \overline{U}\frac{{\left( {\gamma + 2} \right)}}{{R\left( {x,t} \right)}}$$

where $\gamma =9$ gives the blunt velocity profile, as mentioned before. The WSS also depends on the blood viscosity, $\mu =0.032$ (g/cm s) (Colebank et al. 2021), the velocity at the center of the vessel $\overline{U }$ (cm/s), and the dynamic inner radius of the blood vessel wall $R\left(x,t\right)$ (cm^2^). We use the midpoint ($x=L/2$) as the location in the proximal branches for all quantities of interest. We also consider the average pressure, flow rate, and WSS in the distal vasculature in our uncertainty quantification analysis, which corresponds to the zeroth frequency, ${\omega }_{k}=0$. The WSS in the distal vasculature at ${\omega }_{k}=0$ is equivalent to the Poiseuille derived shear stress

32 $${\text{WSS}}_{{{\text{dist}}}} = \frac{{4\mu \overline{Q} }}{{\pi \overline{R}^{3} }}$$

where $\overline{Q }$ and $\overline{R }$ are the average flow rate and radii of the distal vasculature corresponding to ${\omega }_{k}=0$. Lastly, the cyclic stretch (CS) in both the proximal and distal vasculature is calculated as

33 $${\text{CS}} = \frac{{\max \left( {R\left( t \right)} \right) - \min \left( {R\left( t \right)} \right)}}{{\min \left( {R\left( t \right)} \right)}}$$

Though proximal pressure and flow rate are quantities typically studied, WSS and CS are known to affect and regulate cell signaling at the endothelial and smooth muscle cell level (Allen et al. 2023). These mechanotransduction stimuli are rarely examined in modeling studies (Bartolo et al. 2022), though they provide insight into the magnitude of hemodynamic forces in the vasculature and can help guide experimental design.

Lastly, we investigate how uncertainties in the model affect wave-transmission in the proximal arteries and veins using wave intensity analysis (WIA) (Qureshi and Hill 2015; Feng et al. 2021). In short, WIA separates pulse waves within the circulation into forward and backward waves. These waves are further defined as forward compression waves (FCWs, increasing pressure, increasing velocity), forward expansion waves (FEWs, decreasing pressure, decreasing velocity), backward compression waves (BCWs, increasing pressure, decreasing velocity), and backward expansion waves (BEWs, decreasing pressure, increasing velocity). Wave types are hypothesized to correlate with right ventricular dysfunction and pulmonary vascular disease (Su et al. 2016). The classification of each wave type is identical to the analysis presented by (Feng et al. 2021). Though WIA has been used to understand pulmonary arterial hemodynamics (Quail et al. 2015; Qureshi and Hill 2015; Su et al. 2016), the use of WIA in the pulmonary venous system is less common (Mynard and Smolich 2015; Feng et al. 2021).

### Parameter uncertainty and study design

To account for uncertainties and use the PCE framework, we impose uncertainty bounds and prior distributions for our parameters. We consider the following parameters that describe the proximal and distal vasculature:

34 $${\varvec{\theta}} = \left\{ {K_{A} ,K_{ST} ,K_{V} ,\alpha ,\beta ,\ell_{rr}^{A} ,\ell_{rr}^{V} ,r_{{{\text{min}}}} } \right\}$$

The first three parameters describe the material properties of the vasculature, while the latter five describe the structured tree geometry.

We assume that the above parameters have a uniform prior distribution, ${\theta }_{i}\sim \mathcal{U}\left(a,b\right)$, where $a$ and $b$ denote the upper and lower bounds of the parameters. Parameters are scaled to the interval $\left[-\text{1,1}\right]$ during the PCE construction process. A list of the parameters, their upper and lower bounds, and references where applicable can be found in Table 2. The uniform prior distribution in the parameters requires the use of orthogonal Legendre polynomials for the PCE basis functions, $\psi \left(\theta \right)$, as discussed earlier. We compare degree $\mathcal{K}=2, 3,$ and 4 polynomials like previous studies using the 1D framework (Huberts et al. 2014). We assess the PCE accuracy using the mean square error over the validation data

35 $${\varvec{\varepsilon}}_{{{\text{MSE}}}} = \frac{1}{{N_{val} }}\mathop \sum \limits_{i = 1}^{{N_{val} }} \left( {{\varvec{Z}}_{{\varvec{i}}} - M\left( {{\varvec{\theta}}_{{\varvec{i}}} } \right)} \right)^{2}$$

where ${N}_{val}=100$ is the number of validation datasets. Note that ${{\varvec{\varepsilon}}}_{MSE}$ is a vector reflecting the validation error for all validation data. We report the average errors as a metric of validation accuracy for each PCE. We compute the PCEs, their moments, and the Sobol’ indices of our various outputs using the standard ordinary least squares procedure in the *UQlab* software in MATLAB (Marelli and Sudret 2014).

**Table 2:** Parameter descriptions and uncertainties

Parameter	Representation	Bounds (values)	Bounds (relative to mean value)	References
${K}_{A}$	Proximal arterial stiffness (g/cm/s^2^)	[5.60e5, 1.04e6]	[0.7,1.3]	(Qureshi et al. 2014; Mynard and Smolich 2015; Feng et al. 2021; Bartolo et al. 2022)
${K}_{ST}$	Structured tree stiffness (g/cm/s^2^)	[1.75e5, 3.25e5]	[0.7,1.3]	(Qureshi et al. 2014; Feng et al. 2021; Bartolo et al. 2022)
${K}_{V}$	Proximal venous stiffness (g/cm/s^2^)	[5.95e5, 1.11e6]	[0.7,1.3]	(Qureshi et al. 2014; Mynard and Smolich 2015; Feng et al. 2021; Bartolo et al. 2022)
$\alpha$	Radius ratio for $\alpha$ daughter (ND)	[0.80, 0.92]	[0.93, 1.07]	(Qureshi et al. 2014; Chambers et al. 2020; Feng et al. 2021; Colebank et al. 2021; Bartolo et al. 2022)
$\beta$	Radius ratio for $\beta$ daughter (ND)	[0.60, 0.70]	[0.92, 1.07]	(Qureshi et al. 2014; Chambers et al. 2020; Feng et al. 2021; Colebank et al. 2021; Bartolo et al. 2022)
${{\ell}}_{rr}^{A}$	Length-to-radius ratio for the arterial side of the structured tree (ND)	[10, 50]	[0.33,1.67]	(Qureshi et al. 2014; Chambers et al. 2020; Feng et al. 2021; Colebank et al. 2021; Bartolo et al. 2022)
${{\ell}}_{rr}^{V}$	Length-to-radius ratio for the venous side of the structured tree (ND)	[10, 50]	[0.33,1.67]	(Qureshi et al. 2014; Feng et al. 2021; Bartolo et al. 2022)
${r}_{min}$	Minimum radius for terminating the structured tree model (cm)	[1e-3,1e-2]	[0.18,1.82]	(Qureshi et al. 2014; Chambers et al. 2020; Feng et al. 2021; Colebank et al. 2021; Bartolo et al. 2022)

## Results

We use PCEs to propagate uncertainties attributed to the model parameters to multiple quantities of interest. In contrast to prior studies, we calculate the uncertainty and parameter influence in both the proximal and distal vasculature, the latter of which has not been analyzed. Parameter importance is quantified through Sobol’ indices, which are readily available after calculating the PCE coefficients. We investigate typical hemodynamic outputs, like blood pressure and flow rate, but also consider the uncertainties and parameter effects on WSS, CS, and WIA.

### Polynomial chaos surrogate

**Fig. 2:**
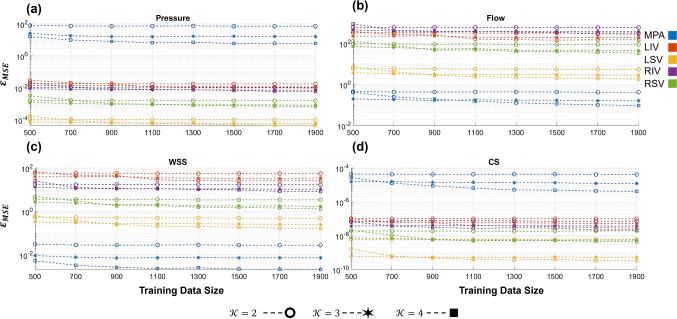
Polynomial chaos expansion accuracy for a set of 100 validation datasets for different training dataset sizes and polynomial order ($\mathcal{K})$. Accuracy in the MPA and four large veins is shown for **a** pressure, **b** flow rate, **c** WSS, and **d** CS. Note that the y-axis is presented on a log-scale

The PCE surrogate is constructed using the nonintrusive ordinary least squares regression approach. We investigate the validation error (Eq. (35)) of the PCE using a set of 100 out-of-sample datasets. Figure 2 illustrates the effect of both training set size and polynomial order on the accuracy of the PCE as an emulator. Results are shown for the MPA and the four large pulmonary veins. Recall that MPA flow rate is a boundary condition in the arteries, while left atrial pressure is a boundary condition for the pulmonary veins. As expected, the $\mathcal{K}=4$ polynomial has the best validation accuracy across all four quantities of interest. The difference in accuracy between polynomial orders ($\mathcal{K}=2, 3,$ or $4$) is most apparent for MPA pressure, MPA CS, and pulmonary venous flow rate. There is some improvement with increasing training data, though the polynomial order has a larger effect on the PCE validation accuracy. Given the apparent benefit of using a higher-order polynomial, we use the PCE with $\mathcal{K}=4$ and 2000 training datasets for the remaining results. We also verified that the total-order Sobol’ indices were relatively stable as a function of sample size and polynomial order (results not shown).

### Proximal vascular hemodynamics

**Fig. 3:**
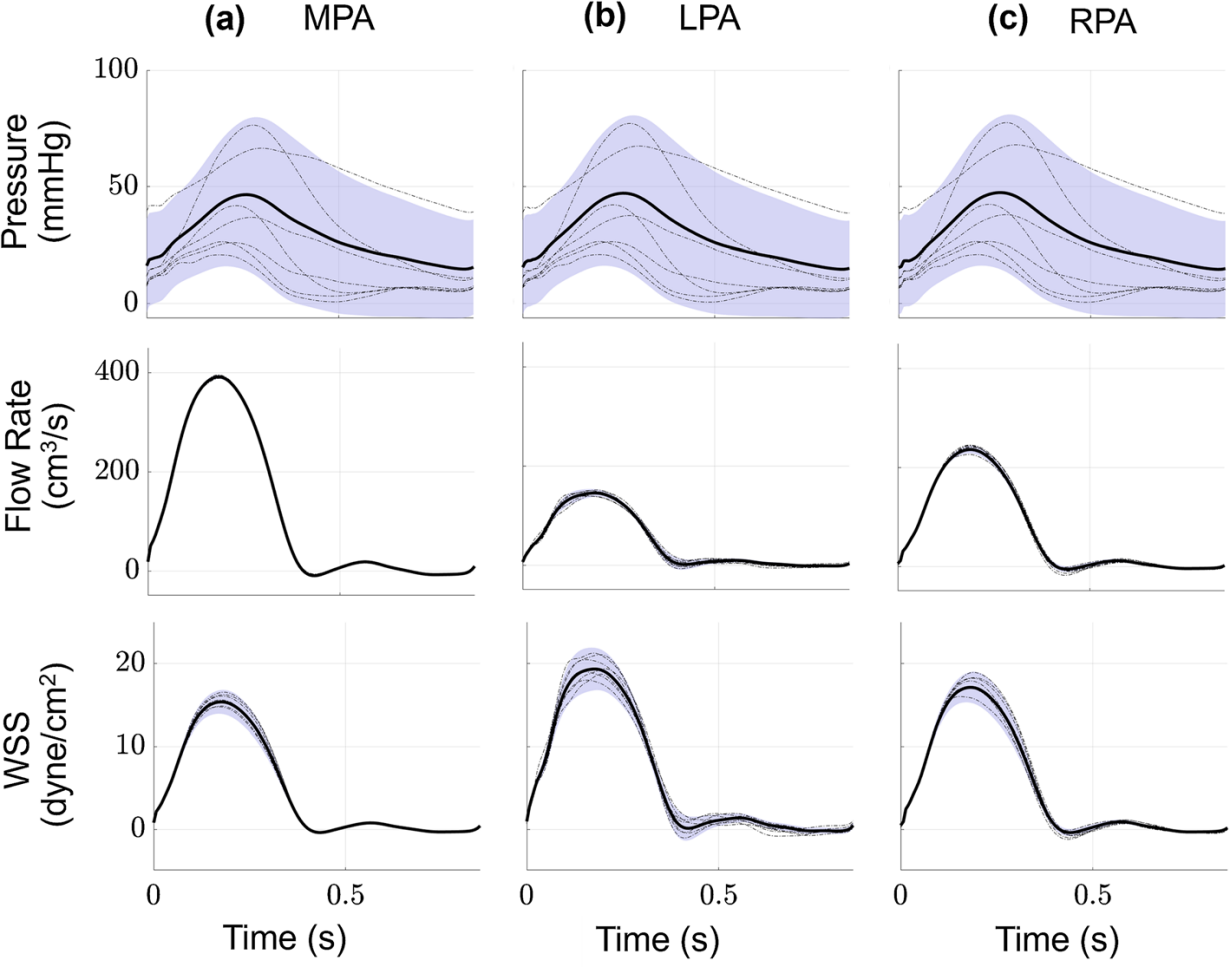
Output uncertainty via the PCEs in the proximal arteries. The average value (black) and one standard deviation from the average (blue) are provided for the **a** MPA, **b** LPA, and **c** RPA. Results show pressure (top row), flow rate (middle row), and WSS (bottom row) uncertainty as a function of time. Realizations from the sampling procedure are shown in dash-dotted lines

The PCE coefficients provide an efficient way to calculate the expectation and variance for each quantity of interest in our model. Figure 3 shows the average pressure, flow rate, and WSS in the MPA as well as the next two arterial branches, the left and right pulmonary artery (LPA and RPA, respectively). We also show one standard deviation from the mean, corresponding to the uncertainty using the PCE coefficients in Eq. (24). The arterial system is driven by a flow profile; hence, flow rate uncertainty, especially in the MPA, is relatively small compared to pressure. Proximal arterial WSS has relatively less uncertainty, with the most variability occurring during peak-systole. The average CS (not shown) is between 4.3 and 4.5% in all the arterial segments, with a standard deviation of 0.25%.

**Fig. 4:**
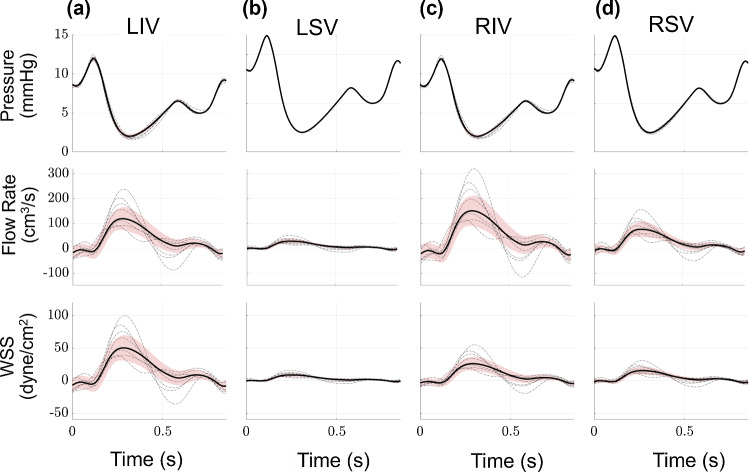
Output uncertainty via the PCEs in the proximal veins. The average value (black) and one standard deviation from the average (red) are provided for the **a** LIV, **b** LSV, **c** RIV, and **d** RSV. Results show pressure (top row), flow rate (middle row), and WSS (bottom row) uncertainty as a function of time. Realizations from the sampling procedure are shown in dash-dotted lines

Proximal vein hemodynamics are coupled to a pressure boundary condition, which leads to relatively small uncertainty in the pressure signals provided in Fig. 4. The dynamics of the pressure signal, corresponding to left atrial reservoir, conduit, and pump function, are correlated with the venous flow rate profile. Flow rate in the pulmonary veins is negligible or slightly negative during the beginning of ventricular contraction, followed by an increase in flow rate while pulmonary venous pressure decreases during atrial relaxation. Pulmonary venous flow rate decreases during the latter phase of the cycle, with a slight notch in flow corresponding to the change in pressure during left atrial filling. Flow into the RPA is greater than the LPA; hence, the flow rate in the right pulmonary venous tree is greater than in the left pulmonary veins. The proximal venous flow rate uncertainty bounds are larger than the arterial side. This subsequently elevates the uncertainty in pulmonary venous WSS, which trends like the flow rate predictions. The LSV flow rate is smaller in magnitude than that of the LIV; thus, the WSS is also smaller in magnitude. Lastly, the venous CS (not shown) is much smaller in the veins relative to the arteries. The average CS across all the proximal veins is between 1.2 and 1.3%, with standard deviation between 0.02 and 0.03%.

### Wave intensity analysis

**Fig. 5:**
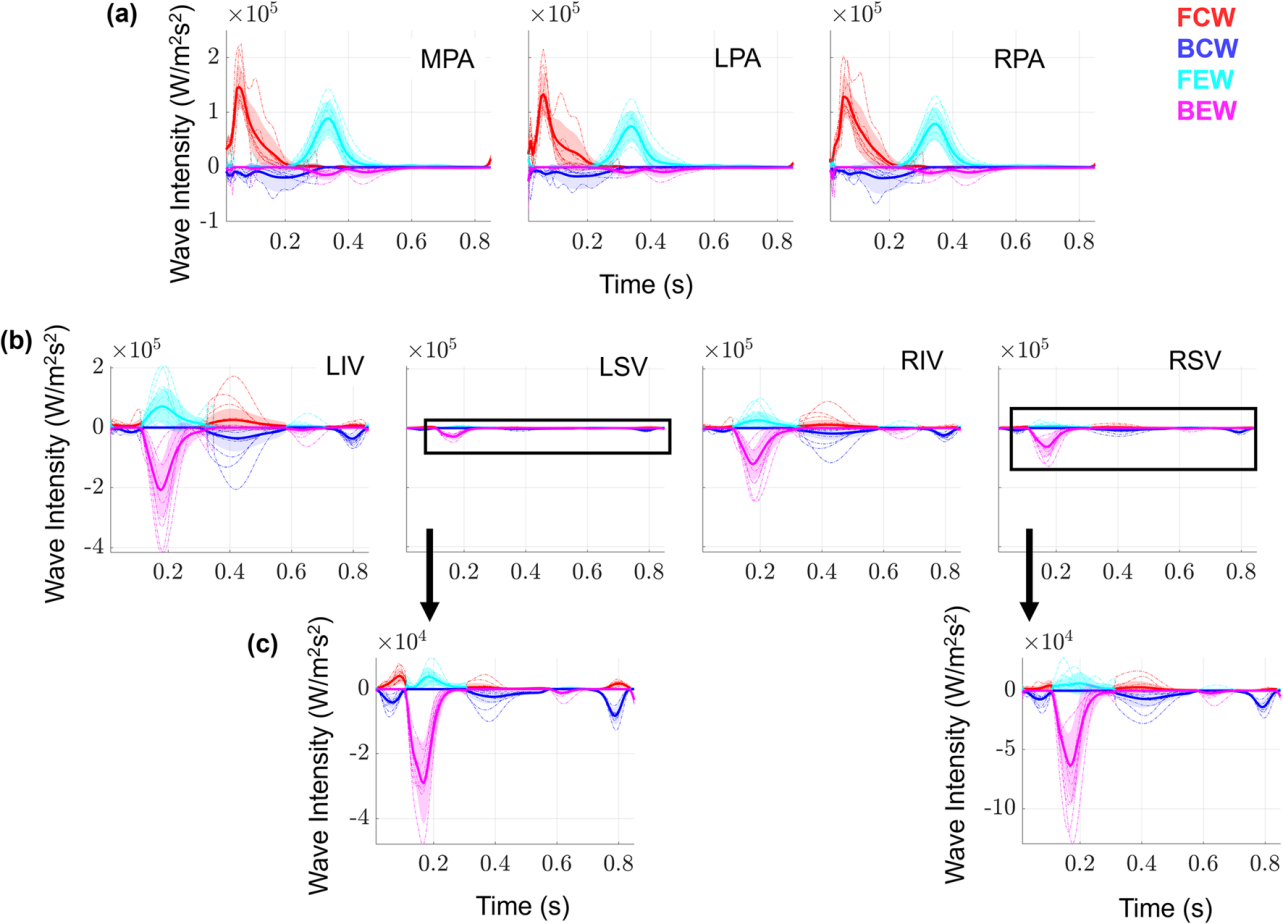
Output uncertainty in wave intensities using PCEs. The average values for FCWs (red), FEWs (cyan), BCWs (blue), BEWs (magenta), and one standard deviation from their respective averages (same colors, shaded) are provided for the **a** first three proximal arteries and **b** the four large veins. Note that, because wave magnitudes vary substantially with vein location, we provide a zoom in subplot in **c** for the LSV, and RSV. Realizations from the sampling procedure are shown in dash-dotted lines

Wave intensities in the proximal arteries and veins are derived from the simulated pressure, flow rate, velocity, and area. The FCWs, which represent increasing pressure and forward flow, occur in the proximal arteries during ventricular ejection, as shown in Fig. 5. There are slight BCWs in the proximal arteries during ejection, but in general these are minimal. Arterial FEWs then follow, representing decreasing pressure and velocity, and then BEWs during decreasing pressure and increasing velocity. These trends are similar in all the proximal arteries, but with decreasing wave magnitudes for branches further down the tree.

The proximal venous WIA results are distinct in their shape and amplitude compared to the arterial results. In general, the proximal veins show a large BEW corresponding to a decrease in pulmonary venous pressure, while flow velocity increases in the venous system. There is also a prominent FEW that occurs simultaneously. All four veins show a relatively large BCW at the end of the cardiac cycle, consistent with the start of atrial contraction and increasing pulmonary venous pressure. On average, both the LIV and the RIV have larger magnitude BEW than the LSV and RSV, consistent with the higher flow rate magnitudes shown in Fig. 4. Individual simulated wave components (shown as dotted lines) vary dramatically in magnitude and in timing. Pulmonary venous wave intensities vary in shape along the venous tree, with the LIV, the RIV, and their first daughter branches (LIV D1 and RIV D1, respectively) exhibiting the largest magnitude for all four wave types.

### Proximal vessel sensitivity

**Fig. 6:**
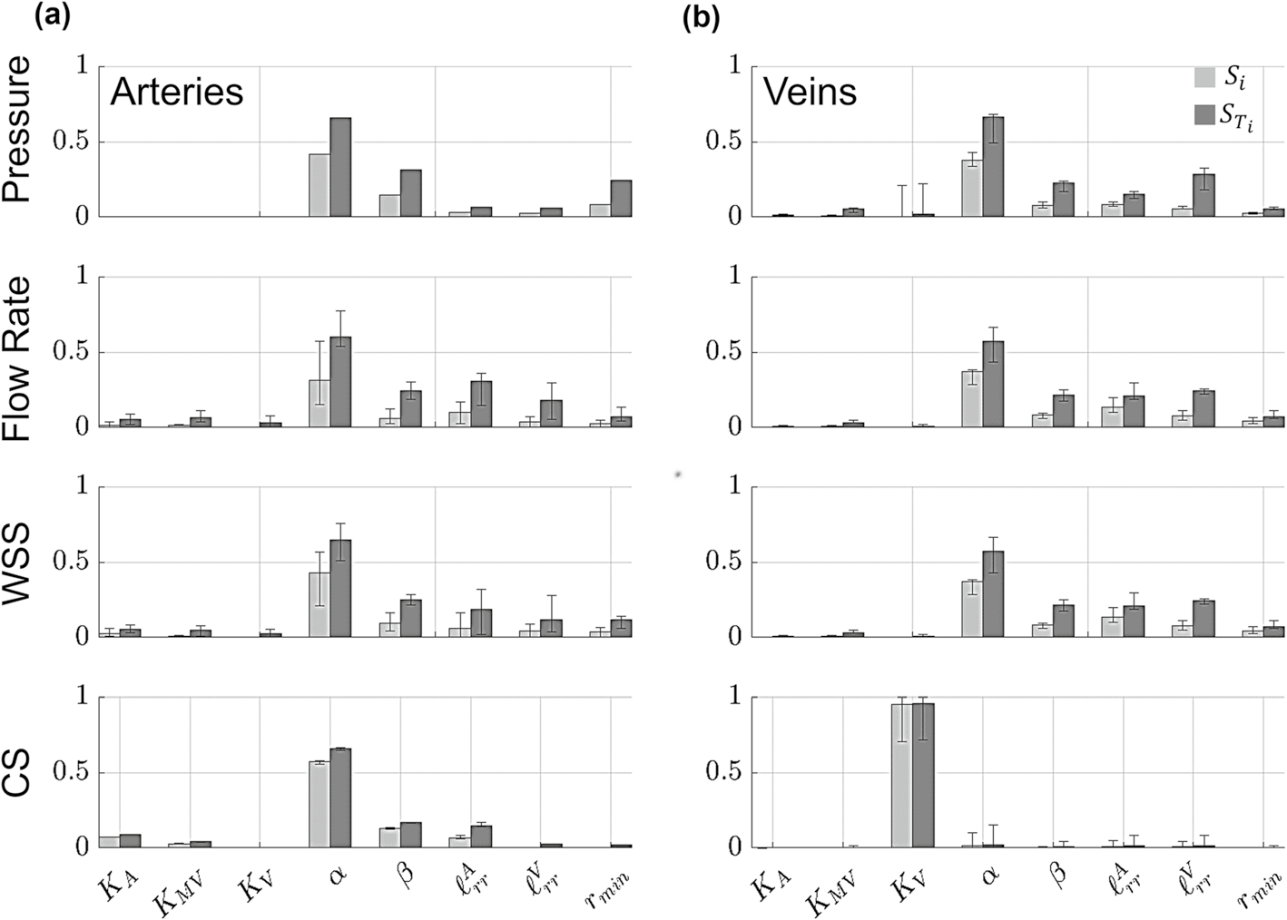
Generalized Sobol’ indices (Eq. (30)) calculated using the PCE coefficients for pressure, flow rate, WSS, and CS. Both first-order (${S}_{i}$, light gray) and total-order (${{S}_{T}}_{i},$ dark gray) Sobol’ indices are provided in the **a** proximal arteries and **b** proximal veins. Each bar height represents the median Sobol’ index for the proximal arteries or veins, while the error bars denote the range of Sobol’ indices found in either proximal vasculature

**Fig. 7:**
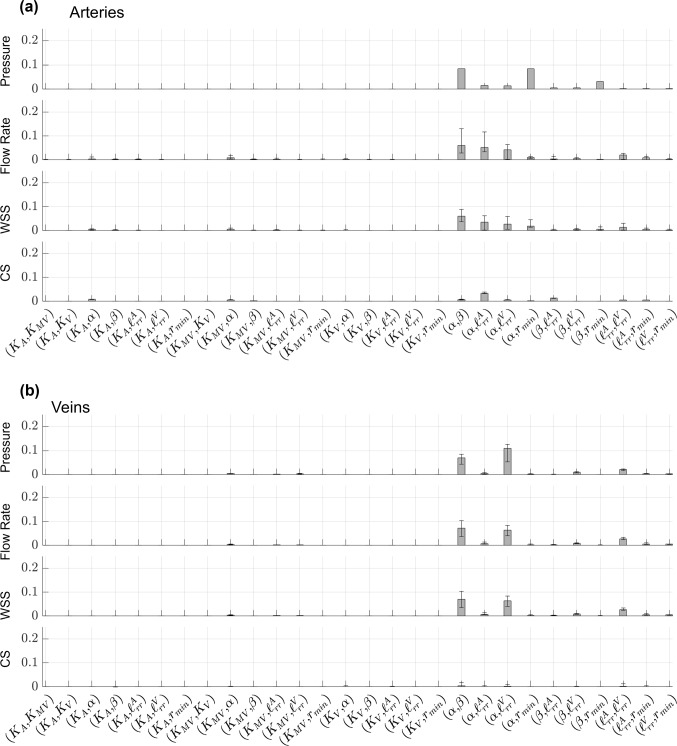
Generalized, second-order Sobol’ indices calculated using the PCE coefficients for pressure, flow rate, WSS, and CS. Values of ${S}_{ij}$ are provided in the **a** proximal arteries and **b** proximal veins. Each bar height represents the median Sobol’ index for the proximal arteries or veins, while the error bars denote the range of Sobol’ indices found in either proximal vasculature

**Fig. 8:**
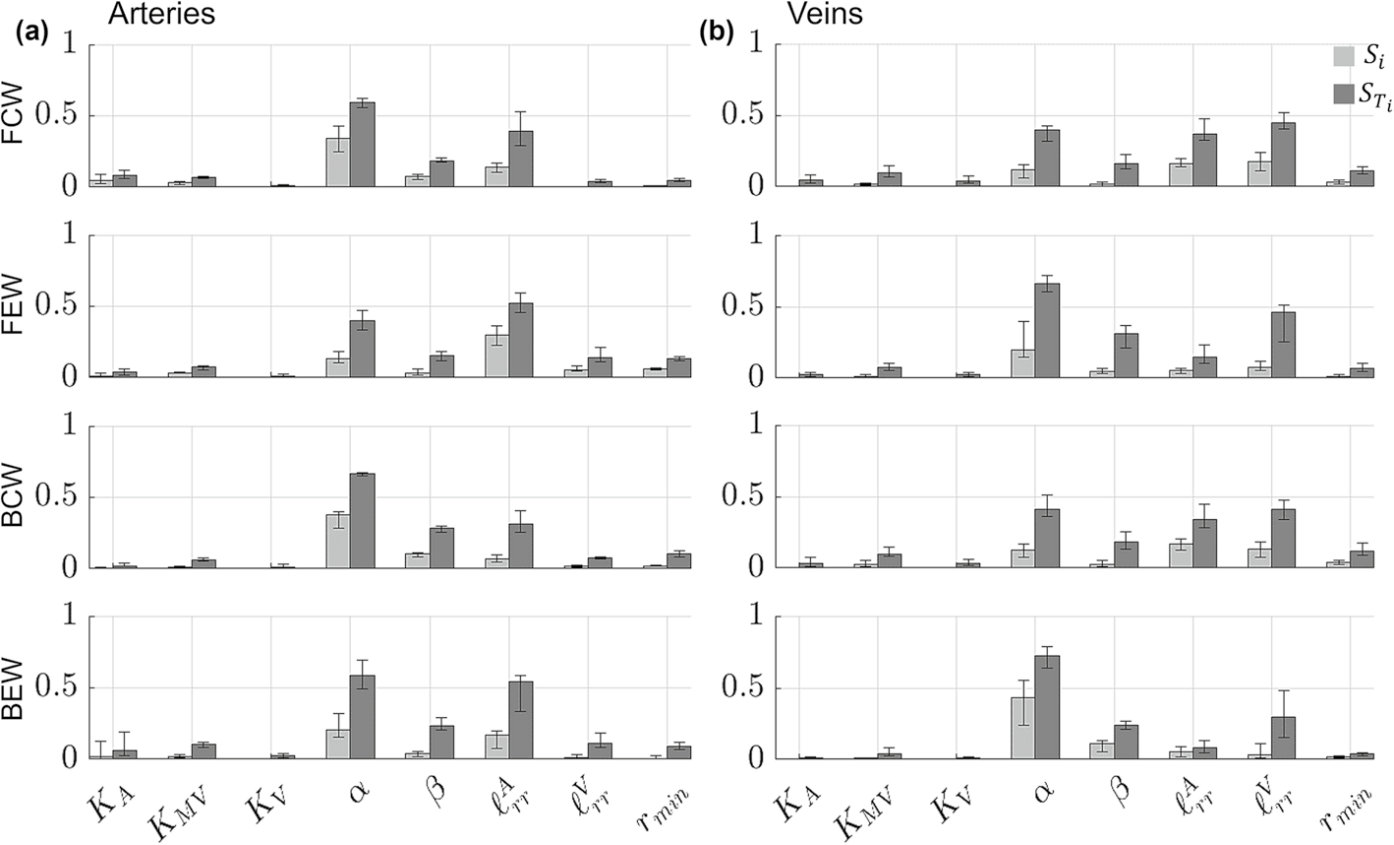
Generalized Sobol’ indices (Eq. (30)) calculated using the PCE coefficients for FCWs, FEWs, BCWs, and BEWs. Both first-order (${S}_{i}$, light gray) and total-order (${{S}_{T}}_{i},$ dark gray) Sobol’ indices are provided in the **a** proximal arteries and **b** proximal veins. Each bar height represents the median Sobol’ index for the proximal arteries or veins, while the error bars denote the range of Sobol’ indices found in either proximal vasculature

**Fig. 9:**
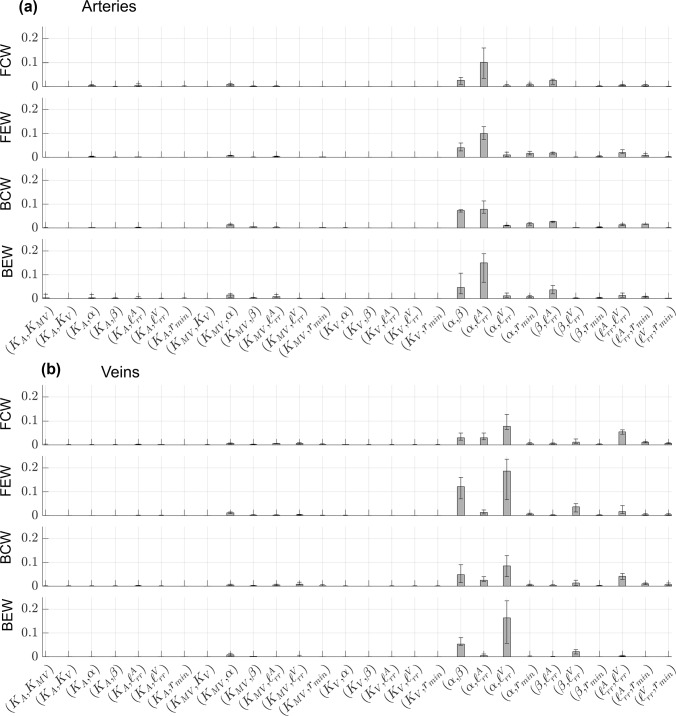
Generalized, second-order Sobol’ indices for FCWs, FEWs, BCWs, and BEWs. Values of ${S}_{ij}$ are provided in the **a** proximal arteries and **b** proximal veins. Each bar height represents the median Sobol’ index for the proximal arteries or veins, while the error bars denote the range of Sobol’ indices found in either proximal vasculature

The coefficients of the PCE allow for straightforward computation of the first-order $(S_{i} )$, second-order $(S_{ij} )$, and total-order $(S_{{T_{i} }} )$ Sobol’ indices. The median Sobol’ indices and range of values for all of the proximal arteries and all of the proximal veins are provided in Figs. 6, 7, 8, and 9, along with error bars representing the range of Sobol’ indices for all the arterial or venous branches.

The values of both ${S}_{i}$ and ${{S}_{T}}_{i}$ are nearly identical for all proximal arteries, as indicated by the negligible error bars in Fig. 6. The structured tree parameters $\alpha$ and $\beta$ are the most influential parameters, followed by $r_{min}$, $\ell_{rr}^{A}$, and $\ell_{rr}^{V}$. In contrast, the flow rate and WSS Sobol’ indices have more variability, especially the values of $S_{i}$ corresponding to the parameter $\alpha$ and the values of $S_{{T_{i} }}$ for $\ell_{rr}^{A}$ and $\ell_{rr}^{V}$. The sensitivity of CS parallels the results for pressure, with the exception that the stiffness parameters $K_{A}$ and $K_{ST}$ are more influential for CS than pressure. In general, the sensitivity indices for pressure and CS are consistent across all of the proximal arteries. There is a notable difference between $S_{i}$ and $S_{{T_{i} }}$ for the parameters $\alpha ,\beta ,\ell_{rr}^{A}$, and $\ell_{rr}^{V}$, which is attributed to higher-order interactions. The second-order indices, shown in Fig. 7, provide evidence that the parameter pairs $\left( {\alpha ,\beta } \right), \left( {\alpha ,\ell_{rr}^{A} } \right),\left( {\alpha ,\ell_{rr}^{V} } \right),\left( {\alpha ,r_{min} } \right),\left( {\beta ,r_{min} } \right)$, and $\left( {\ell_{rr}^{A} ,\ell_{rr}^{V} } \right)$ have non-negligible $S_{ij}$ values.

For the proximal veins, the largest values of ${{S}_{T}}_{i}$ for pressure coincide with the parameters $\alpha , {{\ell}}_{rr}^{V}$, and $\beta$, while there is variability for both ${S}_{i}$ and ${{S}_{T}}_{i}$ for the parameter ${K}_{V}$. The sensitivity of venous flow rate and WSS is like the results found on the arterial side, with less variability in the values of ${S}_{i}$ and ${{S}_{T}}_{i}$. Pulmonary venous CS is almost completely determined by values of ${K}_{V}$, with the other parameters in the system providing little, if any, effects on venous CS. There is a similar difference in ${S}_{i}$ and ${{S}_{T}}_{i}$ values in the venous tree. The second-order indices in Fig. 7(b) show a similar trend to the arterial tree, with a relatively larger interaction effect for $\left({{\ell}}_{rr}^{A},{{\ell}}_{rr}^{V}\right)$.

The median Sobol’ indices corresponding to the four WIA wave types are provided in Fig. 8 and Fig. 9 along with error bars as described in Fig. 6. In general, all four wave types in both the arterial and venous trees are most sensitive to the value of $\alpha$ in the structured tree model. Interestingly, the parameters ${{\ell}}_{rr}^{A}$ and ${{\ell}}_{rr}^{V}$ are second most influential for the arterial and venous branches, respectively, followed by the parameter $\beta$. The value of ${r}_{min}$ has some influence on all four wave types, while the three stiffness parameters are relatively less influential and vary in their effects on the different wave types. Similar to the results in Fig. 6, the values of ${S}_{i}$ are smaller in magnitude than ${{S}_{T}}_{i}$, suggesting some interactions between parameters. The second-order indices presented in Fig. 9 indicate non-negligible second-order interactions between $\left( {\alpha ,\beta } \right), \left( {\alpha ,\ell_{rr}^{A} } \right),\left( {\alpha ,\ell_{rr}^{V} } \right),\left( {\alpha ,r_{min} } \right),\left( {\beta ,\ell_{rr}^{V} } \right)$, and $\left( {\ell_{rr}^{A} ,\ell_{rr}^{V} } \right)$. In particular, the interactions between $\left( {\alpha ,\ell_{rr}^{A} } \right)$ and $\left( {\alpha ,\ell_{rr}^{V} } \right)$ are the strongest for the proximal arteries and veins, respectively.

### Distal vascular hemodynamics

**Fig. 10:**
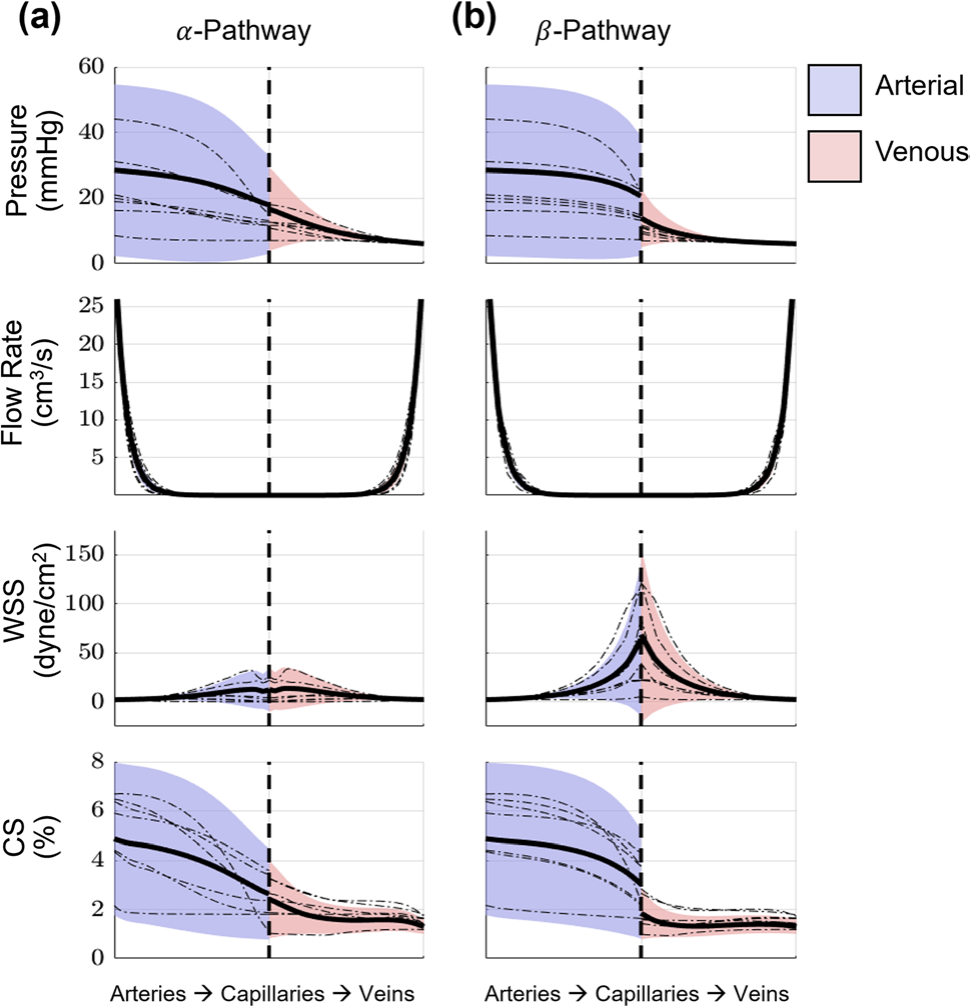
Output uncertainty via the PCEs in the distal arteries and veins of one of the structured tree beds. The average value (black) and one standard deviation from the average (blue or red shade) are provided for the **a** $\alpha$-pathway and **b** $\beta$-pathway. Results show the pressure, flow rate, WSS, and CS uncertainty over the structured tree. Values on the left-most side of the x-axis correspond to the largest arteries in the structured tree, while values on the right-most side of the x-axis correspond to the largest veins in the structured tree. The dashed black line denotes the transition from arteries to veins in the structured tree. Realizations from the sampling procedure are shown in dash-dotted lines

We use the same PCE framework to investigate the uncertainties in the distal vasculature as predicted by the structured tree model. The structured tree model is run for the same model parameters used to generate proximal hemodynamics shown previously. Figure 10 shows the uncertainty in one structured tree (corresponding to the first daughter of the right inferior pulmonary artery and vein, RIA-D1 and RIV-D1, respectively). The other structured tree locations show similar results and are provided in the Supplement. Since the value of ${r}_{min}$ is included in the uncertain parameter set, the terminal radii for the structured tree change with each draw from the prior distribution. Hence, we quantify the uncertainty of the distal vascular hemodynamics as a function of distance from the end of the structured tree, shown in Fig. 10.

The mean pressure is similar in both the $\alpha$ and $\beta$ pathways on the arterial side, whereas the venous $\beta$ pathway exhibits a slightly smaller mean pressure than the corresponding $\alpha$ pathway at the smallest venous branches. The arterial pressure uncertainty is noticeably larger than the venous uncertainty in the structured tree, and the venous uncertainty decreases as predictions move closer to the proximal veins.

The flow rate predictions in both arterial and venous trees appear nearly identical; however, the mean flow rate at the end of the $\alpha$ pathway is on the order of 1e-5, whereas flow rates in the $\beta$ pathway on the order of 1e-4. The standard deviation is small in magnitude, ranging from 2 mL/s at the largest branches to approximately 4e-4 in the smallest branches; however, the coefficient of variance (CoV, the ratio of standard deviation to the mean) increases toward the smaller branches, with CoV $\approx$ 0 at the largest branches and CoV $\approx$ 2 in the smallest branches, suggesting more uncertainty for smaller vessel radii. The uncertainty in the $\beta$ pathway is slightly larger than the $\alpha$ pathway.

The results for arterial and venous WSS vary with the $\alpha$ and $\beta$ pathways. The $\alpha$ pathways show a slight increase in the mean WSS near the capillary bed, whereas the $\beta$ pathway exhibits a more drastic increase in shear stress at the microvascular bed. Like the flow rate, the CoV for WSS is 1.8 at the smallest branches and 0.05 at the proximal arteries and veins in both pathways, again showing more uncertainty in the smaller branches. The mean WSS in the $\alpha$ pathway is roughly 15 dyne/cm^2^ at the capillary beds, whereas the $\beta$ pathway has an average WSS that is between 60 and 65 dyne/cm^2^.

Values of CS vary from 8 to 2% in the arterial beds to 4–1% in the venous beds. Like pressure, CS values are relatively continuous across the structured tree in the $\alpha$ pathway, whereas the $\beta$ pathway shows a slight decrease from the arterial to the venous tree after passing the capillary bed. The CS CoV increases slightly in the arterial branches from approximately 60 to 70% as vessel radii decrease, whereas the CoV for venous CS is approximately 60% in the smallest branches but steadily decreases to approximately 20% at the interface with the proximal pulmonary veins.

### Distal vasculature sensitivity

**Fig. 11:**
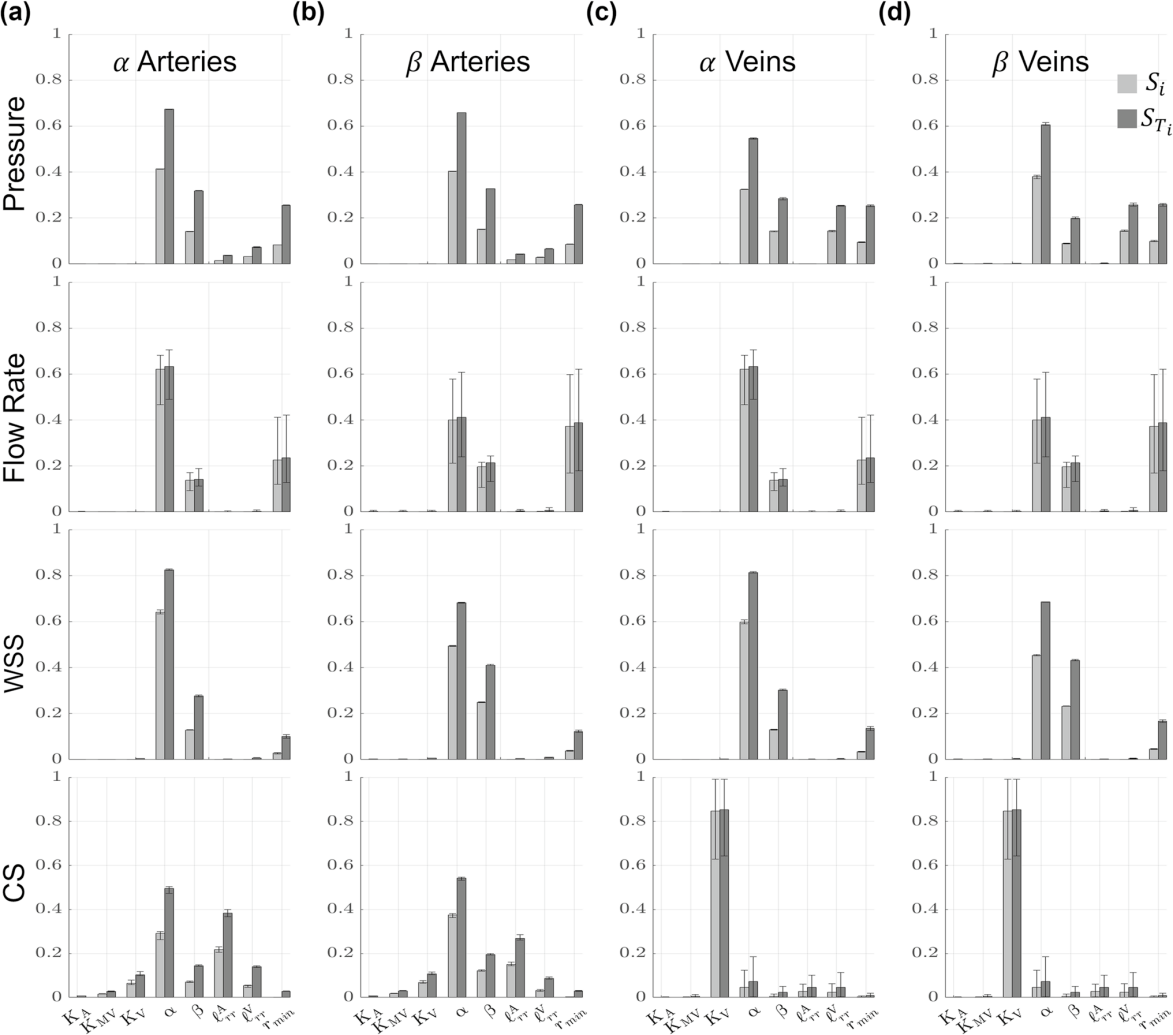
Generalized Sobol’ indices (Eq. (30)) calculated using the PCE coefficients for pressure, flow rate, WSS, and CS across all eight of the structured tree beds. Both first-order (${S}_{i}$, light gray) and total-order (${{S}_{T}}_{i},$ dark gray) Sobol’ indices are provided in the **a** $\alpha$ arteries, **b** $\beta$ arteries, **c** $\alpha$ veins, and **d** $\beta$ veins. Each bar height represents the median Sobol’ index for the distal $\alpha$ and $\beta$ arteries or veins, while the error bars denote the range of Sobol’ indices found in across the different structured tree beds

The PCE coefficients are recomputed for the all the structured tree model predictions in each distal vasculature, corresponding to eight sets of PCE coefficients. Figure 11 shows the median first- and total-order Sobol’ indices and the range of values obtained from all eight sets of structured tree predictions in the arterial and venous $\alpha$ or $\beta$ pathways. The second-order indices, ${S}_{ij}$, are provided in the supplementary material. There is little variability in the pressure sensitivity across the eight structured tree beds. In general, $\alpha$ has the largest ${{S}_{T}}_{i}$ corresponding to the largest influence on pressure. $\beta$ and ${r}_{min}$ are also influential on both arterial and venous pathways. Distinct to the venous trees is the larger pressure sensitivity with respect to ${{\ell}}_{rr}^{V}$. Again, stiffness parameters appear to have a minimal effect on pressure.

The values of ${S}_{i}$ and ${{S}_{T}}_{i}$ for the flow rate vary across the eight structured tree beds, with both $\alpha$ and ${r}_{min}$ exhibiting the largest effects on the flow rate predictions. These two parameters and $\beta$ constitute nearly all of the model sensitivity, with little sensitivity being attributed to the other parameters. In contrast to the other quantities of interest (with the exception of CS, discussed later), the first and total order indices for the flow rate are nearly the same in magnitude for all eight structured tree beds, i.e., ${S}_{i}\approx {{S}_{T}}_{i}$, even though the magnitude of the indices vary with each structured tree bed.

The Sobol’ indices for WSS in the structured tree are similar across the different structured tree beds. Again, $\alpha$ is the most influential parameter, yet the $\beta$ pathway shows a larger sensitivity to the parameter $\beta$ than the $\alpha$ pathways. The parameter ${r}_{min}$ is somewhat influential on all four WSS outputs, while all the stiffness parameters, ${{\ell}}_{rr}^{A}$, and ${{\ell}}_{rr}^{V}$ have little to no effect relative to the other three parameters. The second-order indices for the distal vasculature (shown in the supplement) show substantial second-order interactions between $\alpha , \beta$, and ${r}_{min}$ for WSS predictions in both arterial and venous trees.

Lastly, model predictions of CS vary across all four pathways. The parameters $\alpha , {{\ell}}_{rr}^{A}, \beta ,{{\ell}}_{rr}^{V}$, and ${K}_{V}$ (in order of ${{S}_{T}}_{i}$ magnitude) are the most influential on arterial CS in the $\alpha$ pathway. The arterial $\beta$ pathway is similar but is more sensitive to the $\beta$ parameter. Like the proximal vasculature, the ${S}_{i}$ and ${{S}_{T}}_{i}$ magnitudes for venous CS are largest for the parameter ${K}_{V}$. However, other parameters, such as $\alpha , \beta ,{{\ell}}_{rr}^{A}$, and ${{\ell}}_{rr}^{V}$, are also somewhat influential. The venous structured trees have more variability in ${S}_{i}$ and ${{S}_{T}}_{i}$ values, whereas the arterial sensitivities are more consistent.

## Discussion

Expensive PDE models are difficult to interrogate using traditional sensitivity methods; however, PCEs are a useful emulation tool for this process. Our study identifies the important parameters of a recently established model of the pulmonary arterial and venous circulation (Qureshi et al. 2014; Bartolo et al. 2022) and is the first study to quantify uncertainty in both the proximal and distal vasculature in a spatially multiscale model. We perform a novel analysis on two important vascular mechanical stimuli: WSS and CS. These latter two outputs are important *in vitro* studies, yet they cannot be directly measured preclinically (i.e., in animal models in vivo) or clinically. Overall, our results show that the structured parameters of the distal vasculature ($\alpha ,\beta ,{{\ell}}_{rr}^{A},{{\ell}}_{rr}^{V}$, and ${r}_{min}$) are the most influential, whereas the functional parameters describing stiffness (${K}_{A},{K}_{ST}$, and ${K}_{V}$) are minimally influential with the exception of venous CS.

### Proximal vascular uncertainty

Proximal pulmonary arterial hemodynamics are commonly investigated in PH research. While several computational studies have provided predictions of pulmonary arterial hemodynamics (Qureshi et al. 2014; Bordones et al. 2018; Yang et al. 2019), including work by the present authors (Colebank et al. 2021; Bartolo et al. 2022), few groups have critically examined the uncertainty in these predictions. The uncertainty bounds provided in Fig. 3 show that, even with a fixed inflow profile, there can be large uncertainty in proximal arterial pressure and WSS. This degree of uncertainty is larger than that found by (Paun et al. 2020), who quantified *posterior* uncertainty in a 1D pulmonary hemodynamics model for mice. Our pressure variance is much larger, but is attributed to the *prior* uncertainty (e.g., in Table 2), and would be smaller if we were constructing the posterior uncertainty using data.

Our investigation of 1D pulmonary hemodynamic uncertainty using PCEs is the first; however, several studies have used PCEs to explore uncertainties in similar models of the systemic vasculature. Bertaglia et al. (2021) investigated how geometric and material parameters of a systemic 1D hemodynamics model affected output uncertainty, and found that uncertainties in their parameters contributed to $\pm$ 20 mmHg of uncertainty in thoracic aorta pressure predictions. The study by Eck et al. (2017) used PCEs to quantify the uncertainty in a systemic pulse-wave propagation model and showed a large variance ($\approx$ 45 mmHg) in the systolic pressure predictions. Bartolo et al. (2022) quantified the effects different inflow and outflow boundary conditions on a similar 1D model and showed that MPA flow rate had a large effect on WSS. We did not consider uncertainties in the MPA inflow or left atrial pressure boundary condition, yet still observe variability in arterial and venous WSS predictions.

**Fig. 12:**
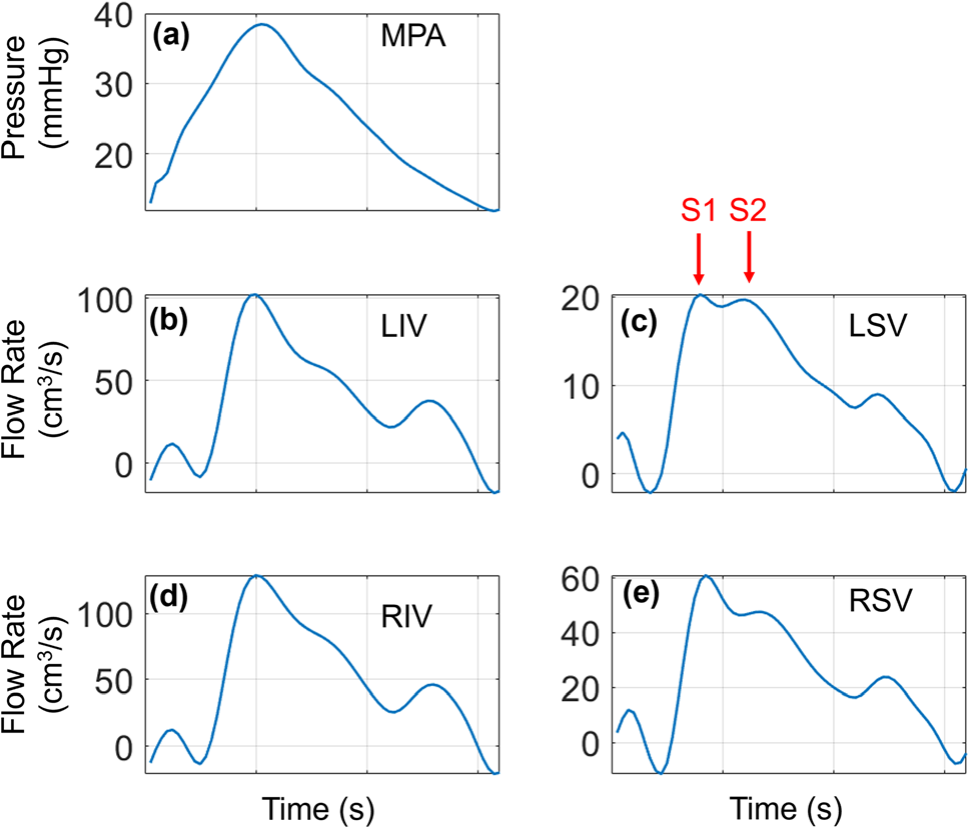
Realization from the training data that includes the “S1” and “S2” components of the pulmonary venous flow rate. **a** MPA pressure; **b** LIV flow rate; **c** LSV flow rate; **d** RIV flow rate; **e** RSV flow rate

Computational models including pulmonary venous hemodynamics are less common than their arterial counterparts. Hellevik et al. (1999) characterized forward and backward waves between the pulmonary veins and left atrium using a three-element transmission line model, with results similar to ours in Fig. 4. While the average pulmonary venous flow rates in Fig. 4 do not exhibit the distinct “S1” and “S2” components of human pulmonary venous flow (Hellevik et al. 1999; Bouwmeester et al. 2014), several of the individual samples generated from our sampling routine, as shown in Fig. 12, do have this feature. Our venous flow rate and WSS values are similar in magnitude to those in Bartolo et al. (2022) but are different in shape due to our dynamic left atrial boundary condition. Feng et al. (2021) coupled a similar 1D hemodynamics model with a 3D model of the mitral valve and left atrium and showed that changes in the parameter ${r}_{min}$ caused changes in LIV flow rate magnitude, consistent with our observed uncertainty in pulmonary venous flow rates.

### Wave intensity

Pulse-wave propagation seen clinically and *in vivo* is driving new research into wave separation and WIA. Only recently has WIA been used to understand the progression of pulmonary vascular disease. The WIA by Quail et al. (2015) showed that time-integrated FCWs, BCWs, and BEWs were significantly different between PH and non-PH groups. The study by Su et al. (2017) used WIA with PAH, chronic thromboembolic pulmonary hypertension (CTEPH), and no PH patients. Su et al. showed that FCW and BCW were elevated in both PH groups and found that the ratio of FCWs to RV contractility distinguished PAH patients from those with CTEPH.

In the absence of detailed data, simulated pressure-flow dynamics can provide WIA results. Mynard and Smolich (2015) modeled a portion of the entire adult circulation and provided WIA results in the pulmonary circulation with similar FCW and FEW magnitudes. In addition, Mynard and Smolich showed predominant BEWs and BCWs in the LIV, consistent with the results shown in Fig. 5. The study by Qureshi and Hill (2015) used a similar two-sided 1D model as we used here and showed minimal backward wave components under normotensive conditions, consistent with a majority of our simulated results.

Relatively few studies, experimental or computational, have considered pulmonary venous WIA. The study by Hellevik et al. (1999) combined clinical data with a Windkessel model and concluded that pulmonary venous waves are driven by left atrial contraction and reflected waves from the pulmonary microcirculation. The preclinical study by Hobson et al. (2007) recorded left atrial and pulmonary venous hemodynamics during acute LV volume loading in anesthetized dogs. They found that left atrial contraction aligned with a prominent, bimodal pulmonary venous BCWs. Our venous WIA results show a similar feature, with one BCW occurring at the middle of the cardiac cycle (during left atrial filling) and one at the end of the cardiac cycle (atrial contraction prior to ventricular systole). The canine study by Bouwmeester et al. (2014) showed similar results, with a spike in wave intensity following mitral valve opening.

Studies by Feng et al. (2021) and Mynard and Smolich (2015) provided pulmonary venous WIA results from a computational model. Both computational studies show a BCW and BEW wave during the start and end of atrial contraction, respectively. Our results in Fig. 5 suggest that BEWs are the largest in magnitude, contrasting these other two modeling studies but corroborating the findings by Bouwmeester et al. (2014). Our individual realizations of WIA in Fig. 5 suggest that the present model can provide an array of wave intensity results and could provide data-specific wave intensity profiles.

### Proximal vascular sensitivity

We efficiently and robustly compute Sobol’ indices using the PCE coefficients (Eck et al. 2016). This study is the first to both (a) conduct a formal sensitivity analysis of the structured tree model, and (b) calculate Sobol’ indices for a 1D model of the pulmonary circulation. Previous studies have performed global sensitivity analyses and calculated Sobol’ indices for models of the systemic circulation. A study by Eck et al. (2015) calculated first-order Sobol’ indices for both amplitude and timing of backward pressure waves with respect to different stiffness parameters along the aortic trunk. The authors found that proximal stiffness parameters were more influential than distal stiffness, while our findings show that hemodynamics and WIA results are mostly affected by the parameters in the structured tree (Figs. 6, 7, 8, 9). However, the stiffness parameters ${K}_{A},{K}_{\text{ST}},$ and ${K}_{V}$ have some effects on both forward and backward waves.

No studies have computed Sobol’ indices for a 1D pulmonary circulation model, but some have conducted other types of sensitivity analysis. The study by Mynard and Smolich (2015) looked at the effects of changing cardiac elastance parameters on wave propagation. They found that RV parameters were most impactful on FCWs and FEWs in the MPA, whereas WIA results in the LIV were more sensitive to changes in left atrial elastance and LV end-diastolic elastance. The studies by Qureshi (Qureshi et al. 2014; Qureshi and Hill 2015) found that stiffness was influential on wave speed and WIA results, but that changes in ${r}_{min}$ had the largest effect on pressure predictions in a similar 1D model. Our results show that ${r}_{min}$ is also more important than stiffness in determining forward and backward wave shapes, and that the other structured tree parameters are most influential on all four wave components. This is consistent with the idea that decreased small vessel density due to distal vessel ‘pruning’ (Rahaghi et al. 2016) is correlated with elevated pulmonary pressures and wave reflections in PH. The study by Olufsen et al. (2012) concluded that their pulmonary arterial circulation model was most sensitive to parameters describing the microvasculature. These findings are consistent with our more formal global sensitivity analysis results and suggest that model sensitivity varies with which circulation (systemic or pulmonary, e.g.) and which components of that circulation are considered. Lastly, our investigation of second-order interactions shows that the structured tree parameters interact with each other to affect hemodynamics and WIA results. Moving forward, these findings may inform future studies that collect data to infer the model parameters from hemodynamic data, i.e., performing parameter estimation.

### Distal vascular uncertainty

Computational models that account for both the proximal and distal hemodynamics are rare but provide more insight into potential mechanisms of disease. Several previous studies have used the structured tree model to predict dynamics in the arterial (Olufsen et al. 2012; Chambers et al. 2020; Colebank et al. 2021) or arterial and venous (Qureshi et al. 2014; Feng et al. 2021; Bartolo et al. 2022) distal vasculature, while others, such as Clark and Tawhai (2018), have used different wave-propagation models. The interaction between the pulmonary microcirculation and the proximal arterial and venous trees is significant in disease progression. Hence, a spatially multiscale model, such as the one presented, can test mechanistic hypotheses regarding the role of microvascular dysfunction in disease development.

The results in Fig. 10 are from a representative structured tree; however, all the structured tree predictions (see the Supplement) are similar in shape and magnitude, with the exception of flow rate. In general, pressure uncertainty is largest at the arterial root of the structured tree and steadily decreases toward the microcirculation and venous trees. The proximal arterial pressure has a standard deviation of 20—35 mmHg, consistent with the standard deviation at the start of the arterial structured tree, which is 20—25 mmHg. These findings suggest a similar uncertainty across these two scales. Pressure uncertainty decreases until reaching the proximal veins, which, due to the left atrial pressure boundary condition, have minimal uncertainty. This again suggests that the uncertainty is communicable across the different scales. The flow rate uncertainty is relatively small in magnitude in all the structured trees. However, the time-averaged flow rate in the proximal veins has small uncertainty (CoV between 2 and 17%), consistent with the smaller standard deviation in the mean flow rate in the structured tree in the venous tree.

The $\alpha$ pathway of the structured tree contains the largest number of branches and the $\beta$ pathway contains the least. Hence, the $\alpha$ pathway will include more generations in the structured tree and have a smaller mean flow rate at the capillary beds. The WSS plots of Fig. 10 show larger magnitudes in the $\beta$ pathway relative to the $\alpha$ pathway. The time-averaged WSS, given by Poiseuille (see Eq. (28)), is dependent on time-averaged flow rate, time-averaged radius, and the radius-dependent viscosity. Since the minimum radii and viscosity values will be similar in both the $\alpha$ and $\beta$ pathways, the biggest contributor to WSS differences is the flow rate magnitude in the two pathways. Lastly, the average CS and its uncertainty decrease from the arterial side to the venous side. The average CS decreases more in the $\beta$ pathway in comparison to the $\alpha$ pathway. The small vessels adhere to a linear pressure-area relationship; hence, pressure and CS (a function of vessel radius) trend in a similar fashion.

Qureshi et al. (2014) used the two-sided 1D model to predict the mean pressure across the $\alpha$ and $\beta$ pathway. The authors also found that reducing the vascular density by 30% elevated mean arterial pressure in the distal vasculature to 50 mmHg, which is within the range of our results in Fig. 10. Bartolo et al. (2022) showed that WSS in the $\beta$ pathway is typically larger in magnitude relative to the $\alpha$ pathway, and that CS values between 10 and 20% in the arterial beds and 10–5% in the venous beds. Our CS values are smaller in magnitude, which can be attributed to the number of branches in our proximal vasculature which decreases the mean flow rate, and hence the stretch, in the structured trees. The results in Fig. 10 provide a starting point for *in vitro* studies investigating the roles of WSS or CS on pulmonary vascular cells. As noted in the review by Allen et al. (2023), these mechanobiological stimuli are hypothesized to progress pulmonary vascular diseases and can be studied in detail only when appropriate stimuli magnitudes have been calculated from *in-silico* or *in vivo* studies.

### Distal vascular sensitivity

The structured tree model contains multiple parameters describing the geometry and material properties of the distal vasculature. Given that the structured tree model is less commonly used than other boundary condition models (e.g., the Windkessel), fewer studies have sought to quantify the impact of the model’s parameters. The distal vascular hemodynamics are on average most sensitive to parameters in the structured tree. Both the median and range of first- and total-order Sobol’ indices (${S}_{i}$ and ${{S}_{T}}_{i}$, respectively) in Fig. 11 show that parameters describing the structured tree geometry are most important, except for ${K}_{V}$ and venous CS.

To date, papers using the structured tree model have only performed informal sensitivity analyses. The study by Qureshi et al. (2014) illustrated that smaller $\alpha$ and $\beta$ induced substantial changes in the mean pressure along the structured tree. The results in Fig. 11 provide evidence that the parameters $\alpha$ and $\beta$, which control the structured tree density, are on average the most influential parameters. In contrast to the proximal vascular results, the parameter ${r}_{min}$ has a larger effect on distal vessel predictions. This is most notable for flow rate, which is the second most influential parameters across $\alpha$ and $\beta$ pathways in both arterial and venous circulations.

Our results support a relationship between microvascular density and pulmonary hemodynamics, which has been documented previously in imaging studies. Gerges et al. (2020) conducted a prospective histological analysis of CTEPH lung biopsies and found that patients with adverse outcomes often had less arterial and venous remodeling than patients who responded well to treatment. Another retrospective histological study by Fayyaz et al. (2018) found that patients diagnosed with PH had more intermediate vessels ($\le$ 100 $\mu$ m) with intimal thickening relative to control. The authors also showed a strong positive relationship between the transpulmonary gradient (the difference between mean pulmonary arterial pressure and pulmonary capillary wedge pressure) and intermediate vessel intimal thickness, suggesting a significant role of the microvasculature in the progression of PH after heart failure. This again suggests that parameters describing small vessel density and geometry are most important to proximal and distal vascular hemodynamics, congruent with our findings here.

### Limitations

Our study conducted a formal sensitivity analysis for a 1D model of pulmonary arterial and venous hemodynamics. We considered uncertainties in proximal vascular stiffness, distal vascular stiffness, and structured tree parameters, but assumed that the arterial, venous, and microcirculation material properties ($Eh/{r}_{0}$) were constant. Prior studies have included radius dependent stiffness (Qureshi et al. 2014; Bartolo et al. 2022), although it is unclear if this is physiological given limited experimental data in the pulmonary vasculature. Our findings show that, even for large stiffness values, the structured tree parameters are still more influential and would not change our current findings. We also assumed that the branching properties of each arterial and venous segment shared common $\alpha$ and $\beta$ values. Given the incredible importance of these parameters, our findings support informing these values from data. Future studies should also investigate whether differences in branching structure give rise to differences in physiological and pathological function.

Our parameter values are reflective of previous studies using similar computational models. However, sex, age, and body surface area affect patient-specific parameters, and these factors also affect the development of PH. For instance, female sex is a known risk factor in the development of PAH, yet female sex is also correlated with higher survival rates and right ventricular resilience than male sex (Ventetuolo et al. 2014). Female sex is also associated with reduced vascular compliance (DesJardin et al. 2024), and, under normotensive conditions, a smaller pulmonary vascular volume than males (John et al. 2023). Age also contributes to pulmonary vascular stiffness and is thought to affect pulmonary endothelial and smooth muscle cell function (Allen et al. 2023). Though these topics are out of scope for the present manuscript, our analysis shows the range of hemodynamic values that likely include both sex and age effects, and will be leveraged in future, patient-specific studies.

We did not consider any uncertainties in the inflow or outlet boundary conditions. Moreover, we assume that the blood velocity in the pulmonary circuit has a constant, blunt shape. While a previous study in canines showed that $\gamma =5$ describes pulmonary arterial flow velocity during systole (Kachabi et al. 2024), the actual blood velocity in the arteries may take on a more Womersley like profile (van de Vosse and Stergiopulos 2011). The blood velocity profile in the venous circulation has not been documented either. Our simulations provide Reynolds numbers supportive of inertially driven flow, but venous velocity and area data can help inform this decision. We anticipate that considering inflow and outlet pressure uncertainty, similar to Brault et al. (2017), will increase the uncertainty in flow rate and pressure predictions at the proximal arteries and veins, respectively. Alternatively, coupling this model to a right ventricle and left atrium would allow for more flexibility in the dynamics of the pulse-wave propagation model; however, this would increase the parameter dimensionality of the problem. Our model terminates at the minimum radius ${r}_{min}$, which ignores the possible effects of the pulmonary capillaries. Follow-up studies should implement a model of the pulmonary capillaries, like Clark and Tawhai (2018), to further identify capillary circulation sensitivity and its parameters’ effects on proximal arterial predictions.

Lastly, we used variance-based global sensitivity analysis, which hinges on several assumptions. One of these assumptions is that the variance of the model outputs is a reasonable representation of the actual output distributions. Alternative methods, like Borgonovo indices or entropy-based sensitivity methods, can be used in combination with variance-based methods to confirm which parameters are influential (Borgonovo 2007). Another limitation is the assumption that parameters are independent in their prior space. A similar variance-based method, derived by Kucherenko et al. (2012), exploits the variance of the output as in Sobol’, but is not bound by the assumption of parameter independence. These alternative methods should be kept in mind and used in future studies.

## Conclusions

This study provides uncertainty quantification and sensitivity analysis results for a spatially multiscale hemodynamics model of the proximal and distal pulmonary arterial and venous circulation. We use PCEs as an efficient tool for uncertainty quantification and analyze the sensitivity of multiple quantities of interest using Sobol’ indices. Our results show that the model framework is flexible, given the large uncertainty bounds in nearly all hemodynamic outputs, and that structured tree parameters are in general the most influential. We provide output uncertainty for standard hemodynamic quantities (pressure and flow rate) and quantify uncertainty in WIA and mechanobiological stimuli. Our investigation into pulmonary wave travel and reflections in the pulmonary circuit suggests a critical role for distal vascular density and structure, supporting current evidence regarding small vessel disease in PH. Our CS and WSS uncertainty bounds are informative for new *in vitro* experimental designs that expose various cell types to stretch and flow. We believe that this in-depth model analysis provides key insight into future studies using the structured tree model for patient-specific simulations.

## Supplementary Information

Below is the link to the electronic supplementary material.

Supplementary file1 (DOCX 23053 KB)

## Footnotes

1 https://simvascular.github.io/clinical/pulmonary.html

## Declarations

### Citation diversity statement

In agreement with the editorial from the Biomedical Engineering Society (BMES) (Rowson et al. 2021) on biases in citation practices, we have performed an analysis of the gender and race of our bibliography. This was done manually, though automatic probabilistic tools exist. We recognize existing race and gender biases in citation practices and promote the use of diversity statements like this for encouraging fair gender and racial author inclusion and identifying gaps in scientific representation.

Our references contain 16% woman(first)/woman(last), 12% man/woman, 16% woman/man, and 56% man/man. This binary gender categorization is limited in that it cannot account for intersex, nonbinary, or transgender people. In addition, our references contain 0% author of color (first)/author of color(last), 5% white author/author of color, 16% author of color/white author, and 79% white author/white author. Our approach to gender and race categorization is limited in that gender and race are assigned by us based on publicly available information and online media. We look forward to future databases that would allow all authors to self-identify race and gender in appropriately anonymized and searchable fashion and new research that enables and supports equitable practices in science.
