# Supplementary material for: Efficient uncertainty quantification in a spatially multiscale model of pulmonary arterial and venous hemodynamics
Source: Biomech Model Mechanobiol. 2024 Jul 29;23(6):1909–31. doi: 10.1007/s10237-024-01875-x (PMC11554845; doi:10.1007/s10237-024-01875-x)
Supplement: Supplementary file 1 — Supplementary file1 (DOCX 23053 KB) [file 10237_2024_1875_MOESM1_ESM.docx]

**Supplementary Material for: Efficient Uncertainty Quantification in a Multiscale Model of Pulmonary Arterial and Venous Hemodynamics**

M. J. Colebank^*^, N.C. Chesler

^1^Edwards Lifesciences Foundation Cardiovascular Innovation and Research Center, and Department of Biomedical Engineering, University of California, Irvine, Irvine, CA, USA

* Corresponding author

Email: [mjcolebank@gmail.com](mailto:mjcolebank@gmail.com) (MJC)

1. **Wave Intensity results**

The sampling strategy for constructing the polynomial chaos expansion (PCE) emulator provides multiple realizations of the quantities of interest. While the expectation and variance for the PCE summarize the results, individual realizations can provide insight into the model flexibility. Below are individual realizations for the forward compression wave (FCW), backward compression wave (BCW), forward expansion wave (FEW), and backward expansion wave (BEW) realizations. Results in the three proximal arteries (Fig. S1) and the four proximal veins (Fig. S2) are shown. Note that the magnitudes vary substantially between the four pulmonary veins.

1. **Structured Tree Uncertainty**

The proximal arterial and venous trees are connected using the structured tree model. The main manuscript provides results from a representative structured tree for the expected value and uncertainty for time-averages pressure, flow rate, wall shear stress (WSS), and cyclic stretch (CS). We provide the results from all eight structured tree beds that connect the arterial and venous geometries. These results are provided for each daughter branch connecting the left inferior, left superior, right inferior, and right superior arterial/venous connections in Fig S3- S10.

1. **Structured Tree Second-Order Sobol’ indicies**

The second-order Sobol’ indices, $S_{ij}$ (see main text), are provided for the four quantities of interest. The boxplots represent the median value and the error bars show the range in Fig S11-S14.


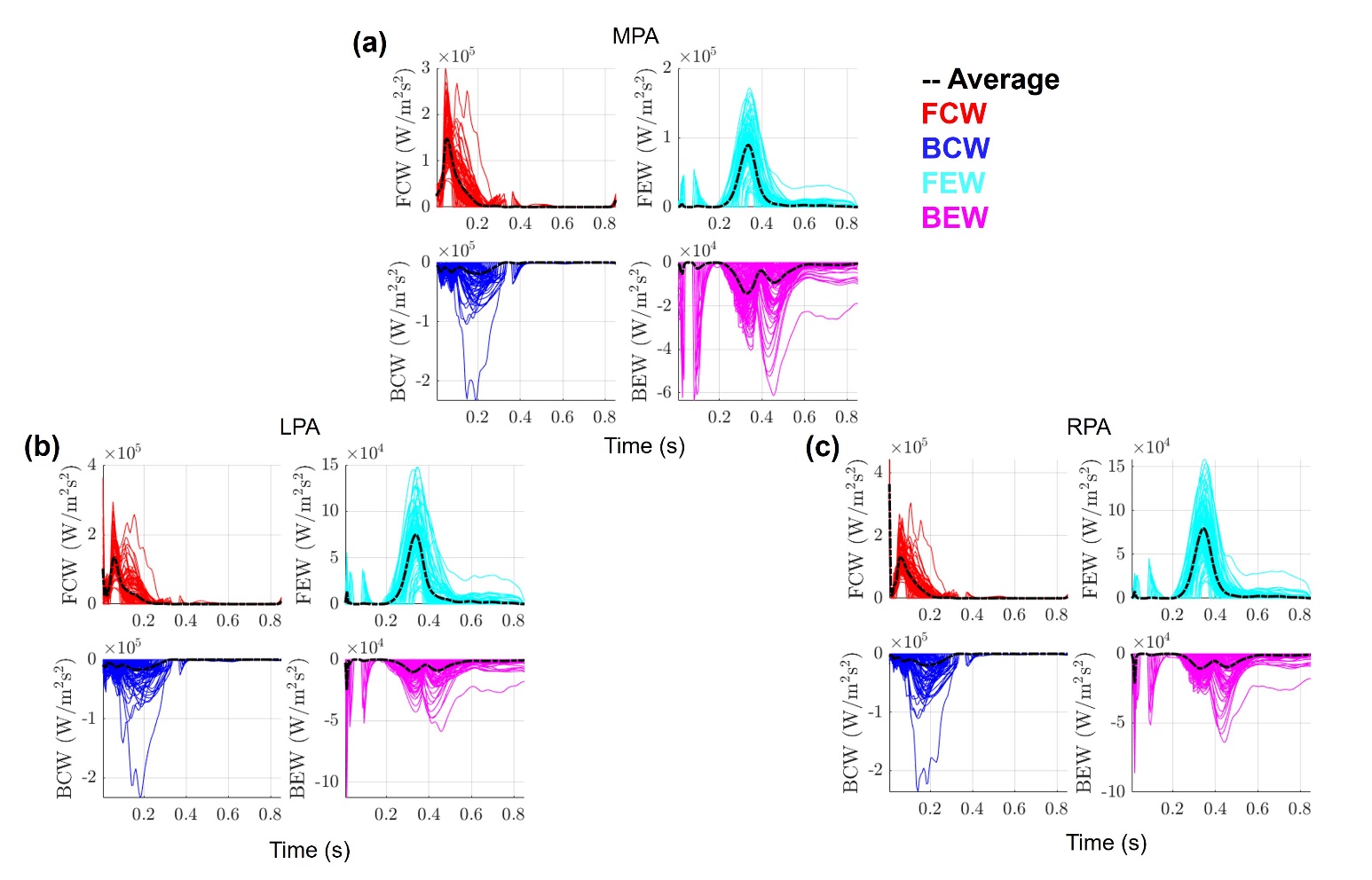


**Fig. S1:** Realizations from random sampling of the parameters in terms of wave intensity components in the three proximal arteries. The individual results are provided in color, while the average is provided as dotted black lines in the main pulmonary artery (MPA) (a), left pulmonary artery (LPA) (b), and the right pulmonary artery (RPA) (c).


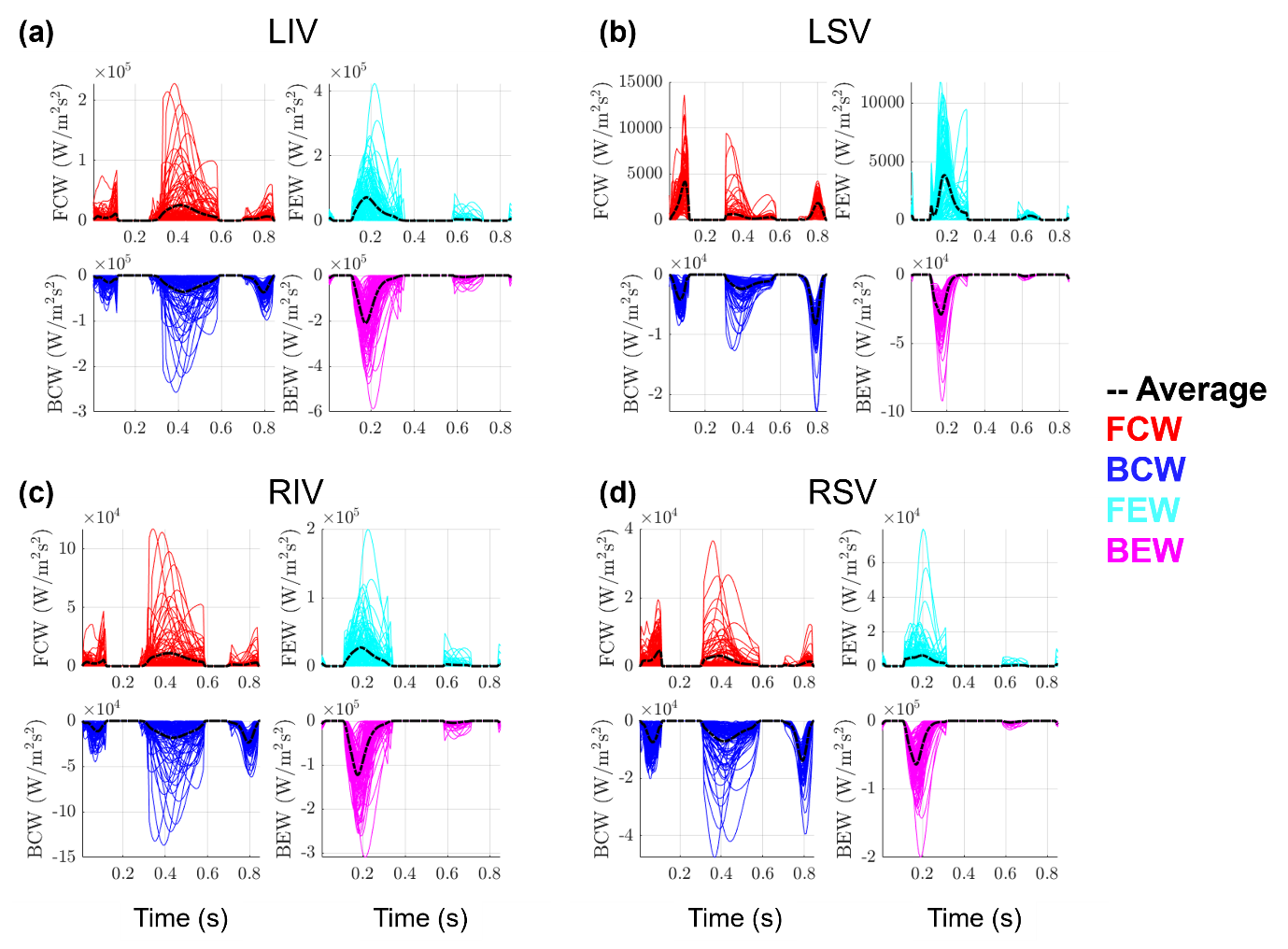


**Fig. S2:** Realizations from random sampling of the parameters in terms of wave intensity components in the four proximal veins. The individual results are provided in color, while the average is provided as dotted black lines. Results are shown in the left inferior vein (LIV) (a), the left superior vein (LSV) (b), the right inferior vein (RIV) (c), and the right superior vein (RSV) (d).


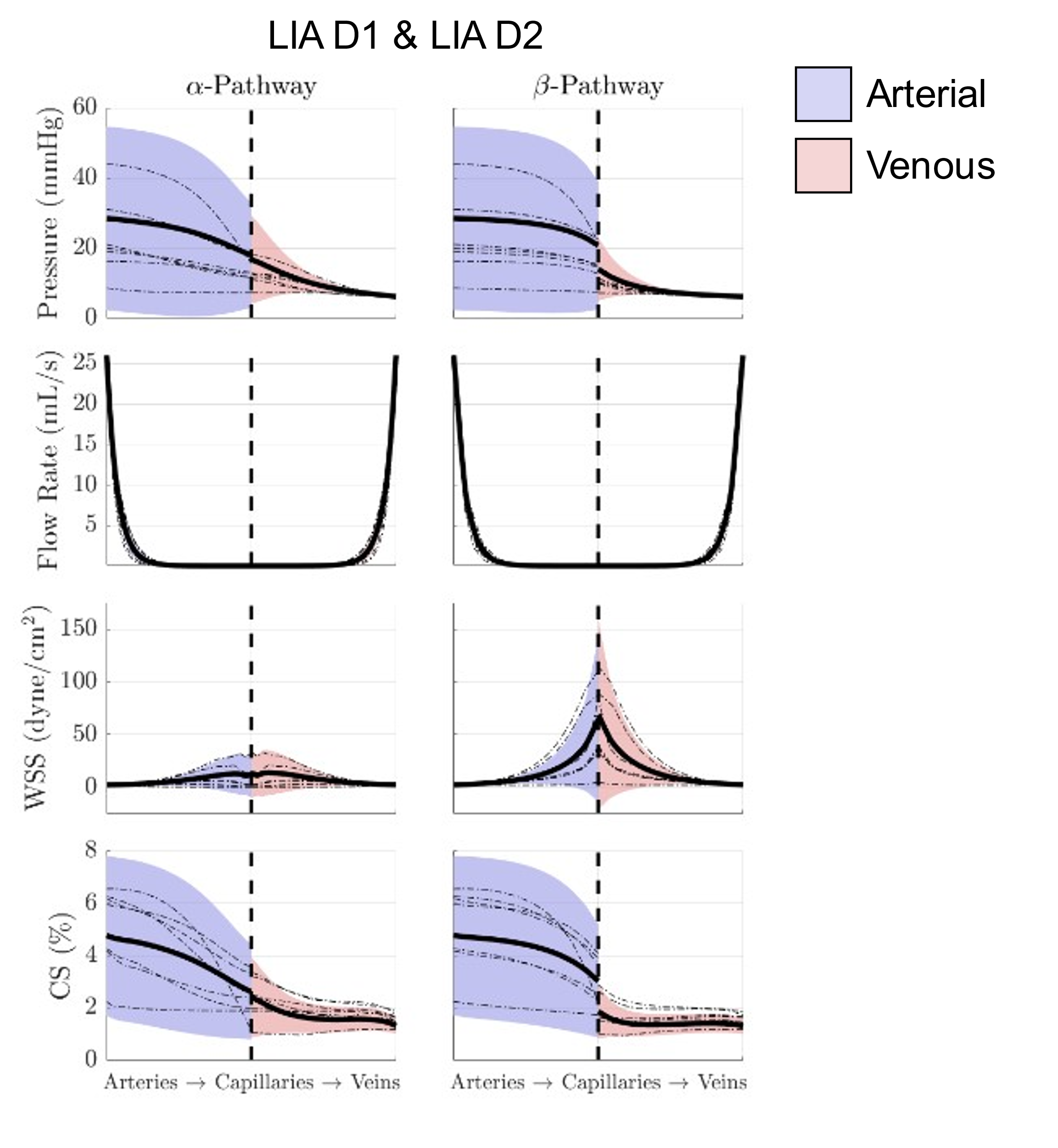


**Fig. S3:** Uncertainty in time-averaged pressure, flow, wall shear stress (WSS), and cyclic stretch (CS) using polynomial chaos expansions. Arterial predictions are provided in blue while venous uncertainty is shown in pink. See main text for more methodological details. LIA: left inferior artery; LIV: left inferior vein. Realizations from the sampling procedure are shown in dash-dotted lines


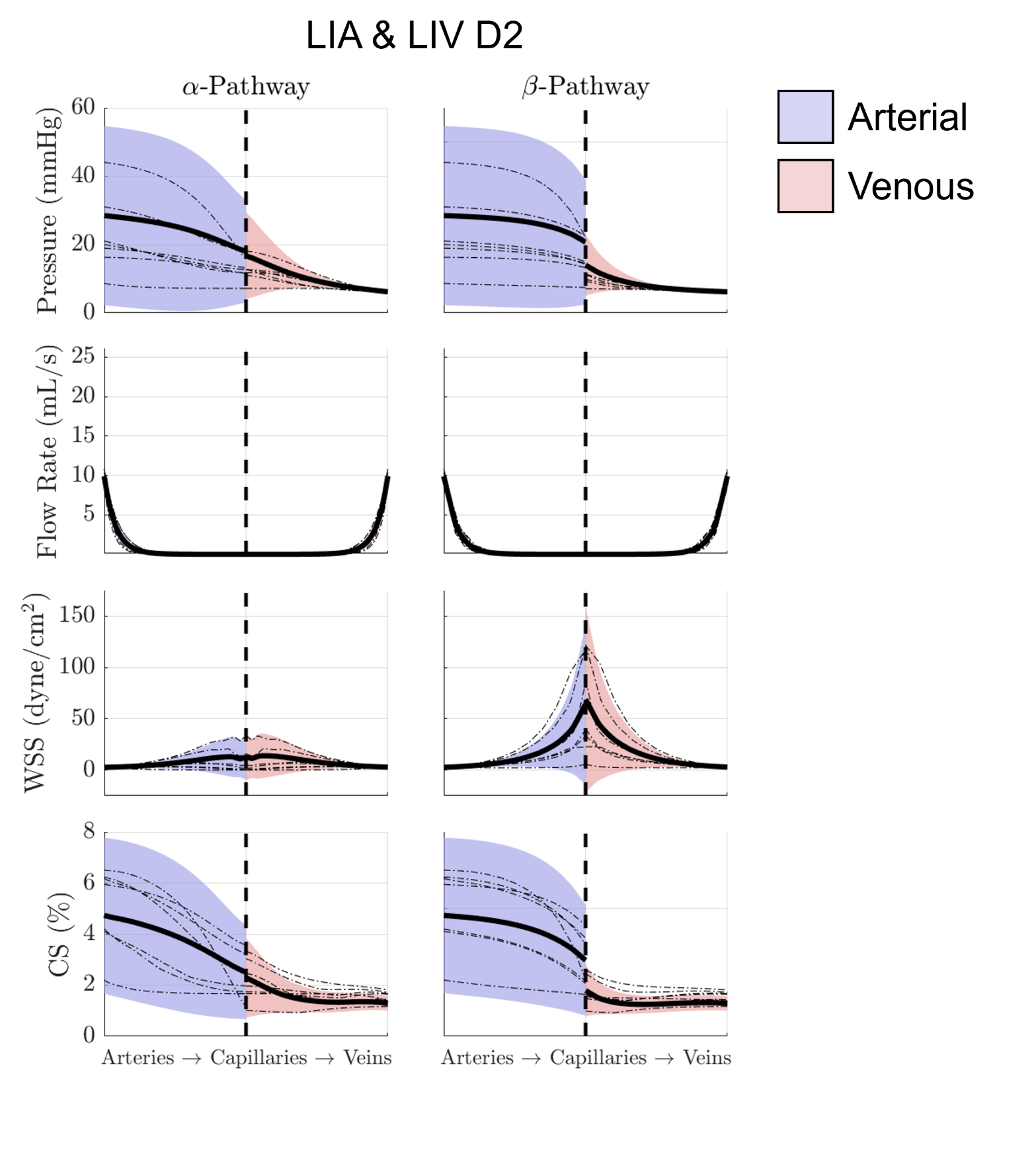


**Fig. S4:** Uncertainty in time-averaged pressure, flow, wall shear stress (WSS), and cyclic stretch (CS) using polynomial chaos expansions. Arterial predictions are provided in blue while venous uncertainty is shown in pink. See main text for more methodological details. LIA: left inferior artery; LIV: left inferior vein. Realizations from the sampling procedure are shown in dash-dotted lines


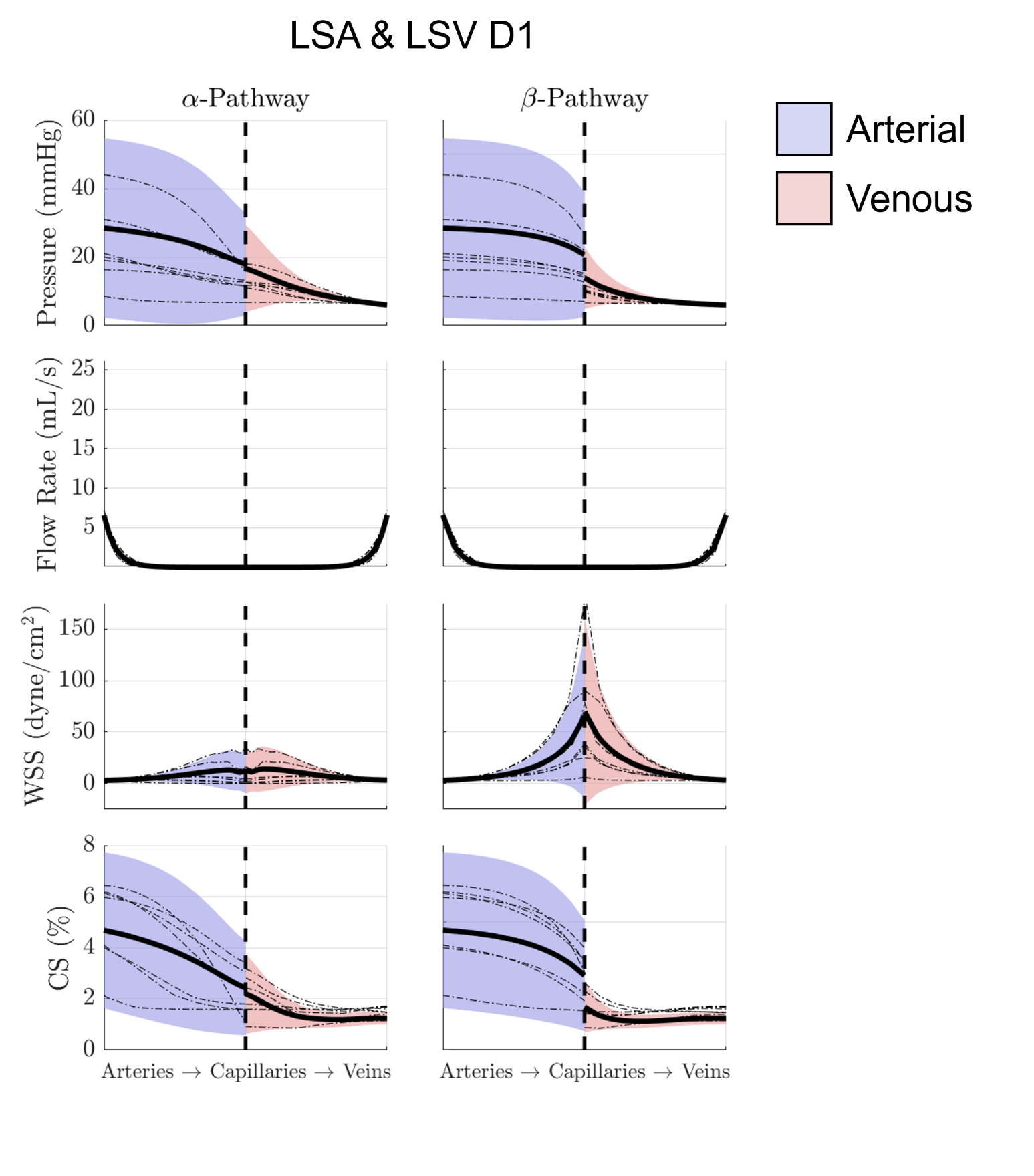


**Fig. S5:** Uncertainty in time-averaged pressure, flow, wall shear stress (WSS), and cyclic stretch (CS) using polynomial chaos expansions. Arterial predictions are provided in blue while venous uncertainty is shown in pink. See main text for more methodological details. LSA: left superior artery; LSV: left superior vein. Realizations from the sampling procedure are shown in dash-dotted lines


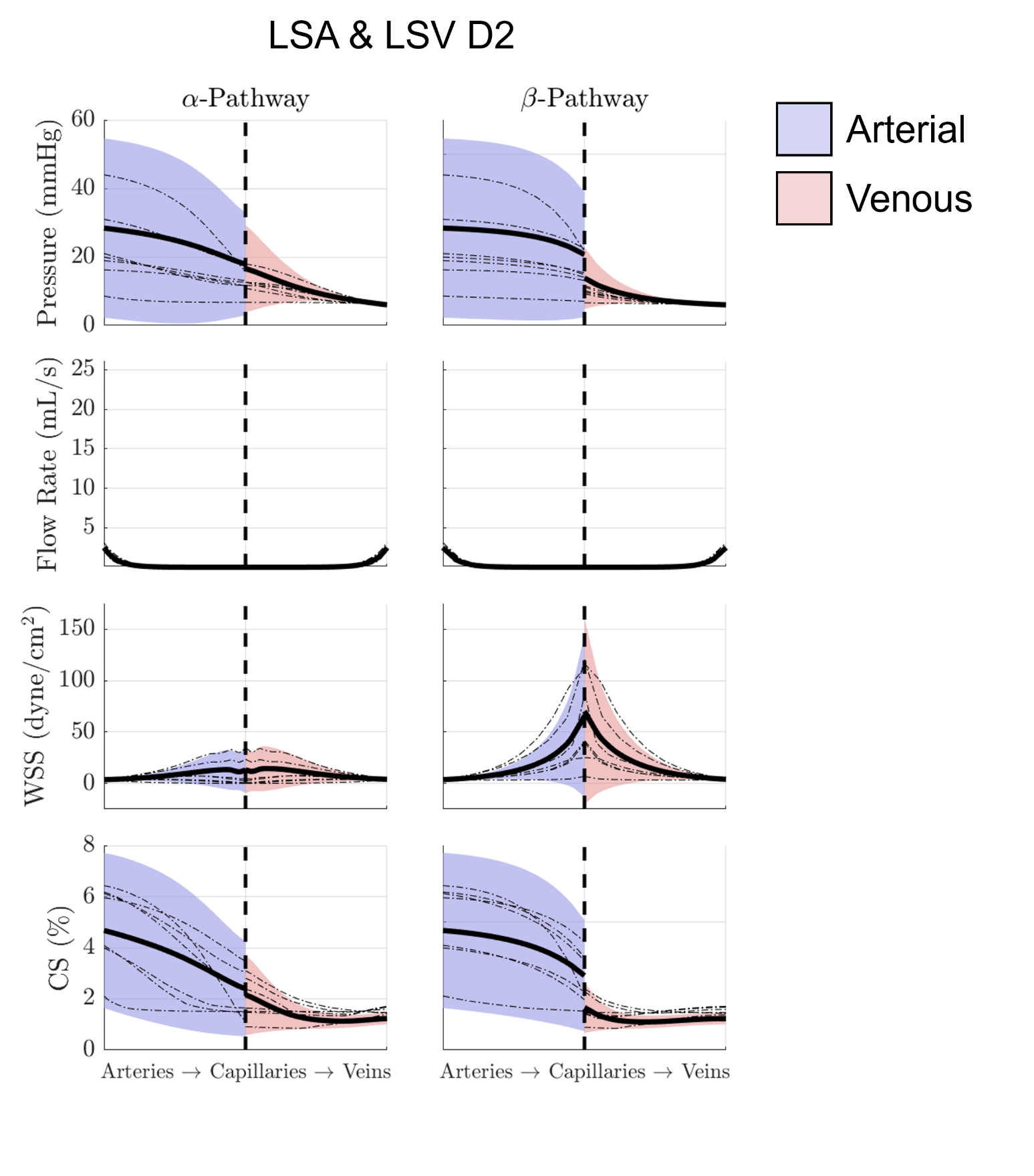


**Fig. S6:** Uncertainty in time-averaged pressure, flow, wall shear stress (WSS), and cyclic stretch (CS) using polynomial chaos expansions. Arterial predictions are provided in blue while venous uncertainty is shown in pink. See main text for more methodological details. LSA: left superior artery; LSV: left superior vein. Realizations from the sampling procedure are shown in dash-dotted lines


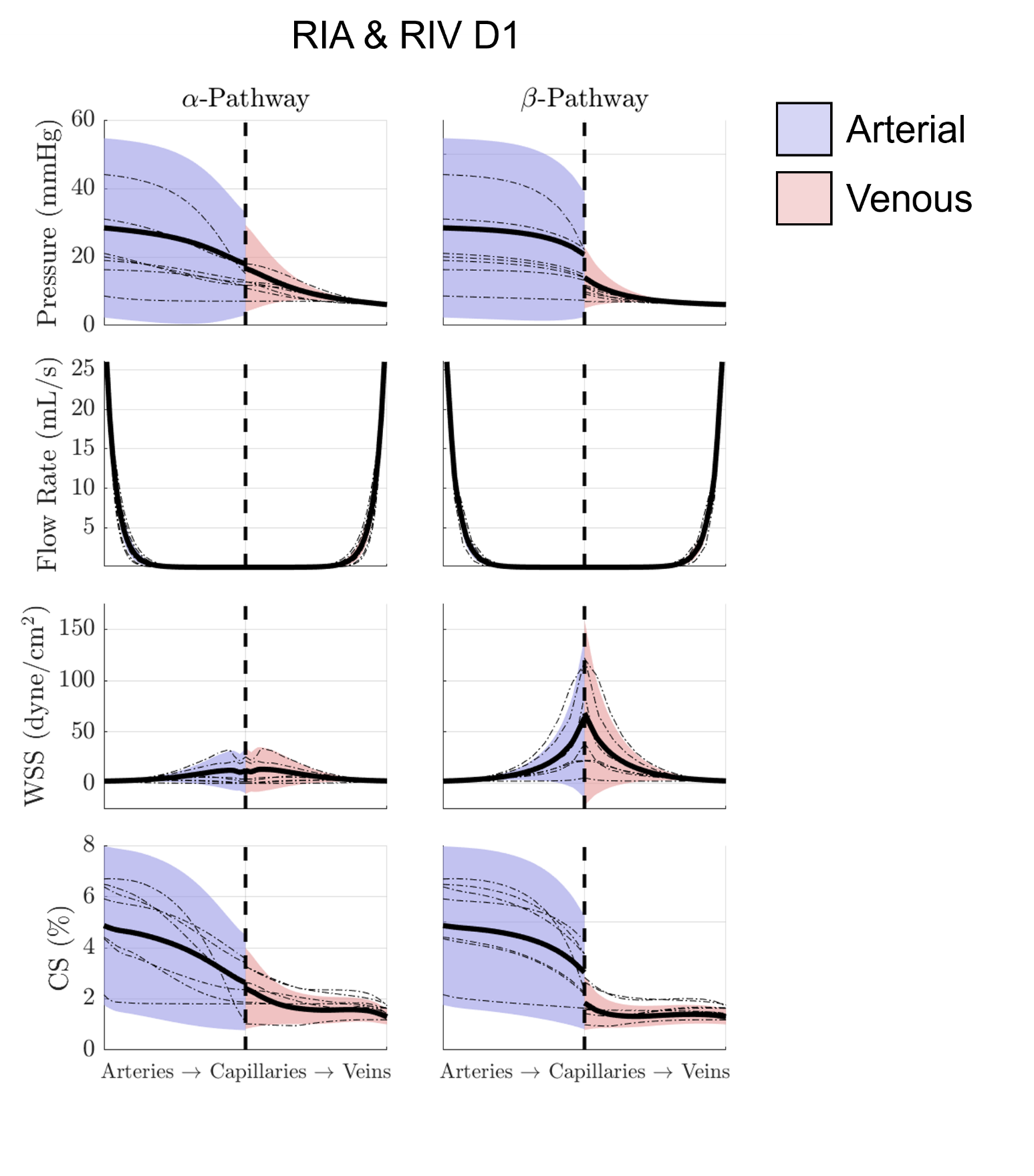


**Fig. S7:** Uncertainty in time-averaged pressure, flow, wall shear stress (WSS), and cyclic stretch (CS) using polynomial chaos expansions. Arterial predictions are provided in blue while venous uncertainty is shown in pink. See main text for more methodological details. RIA: right inferior artery; RIV: right inferior vein. Realizations from the sampling procedure are shown in dash-dotted lines


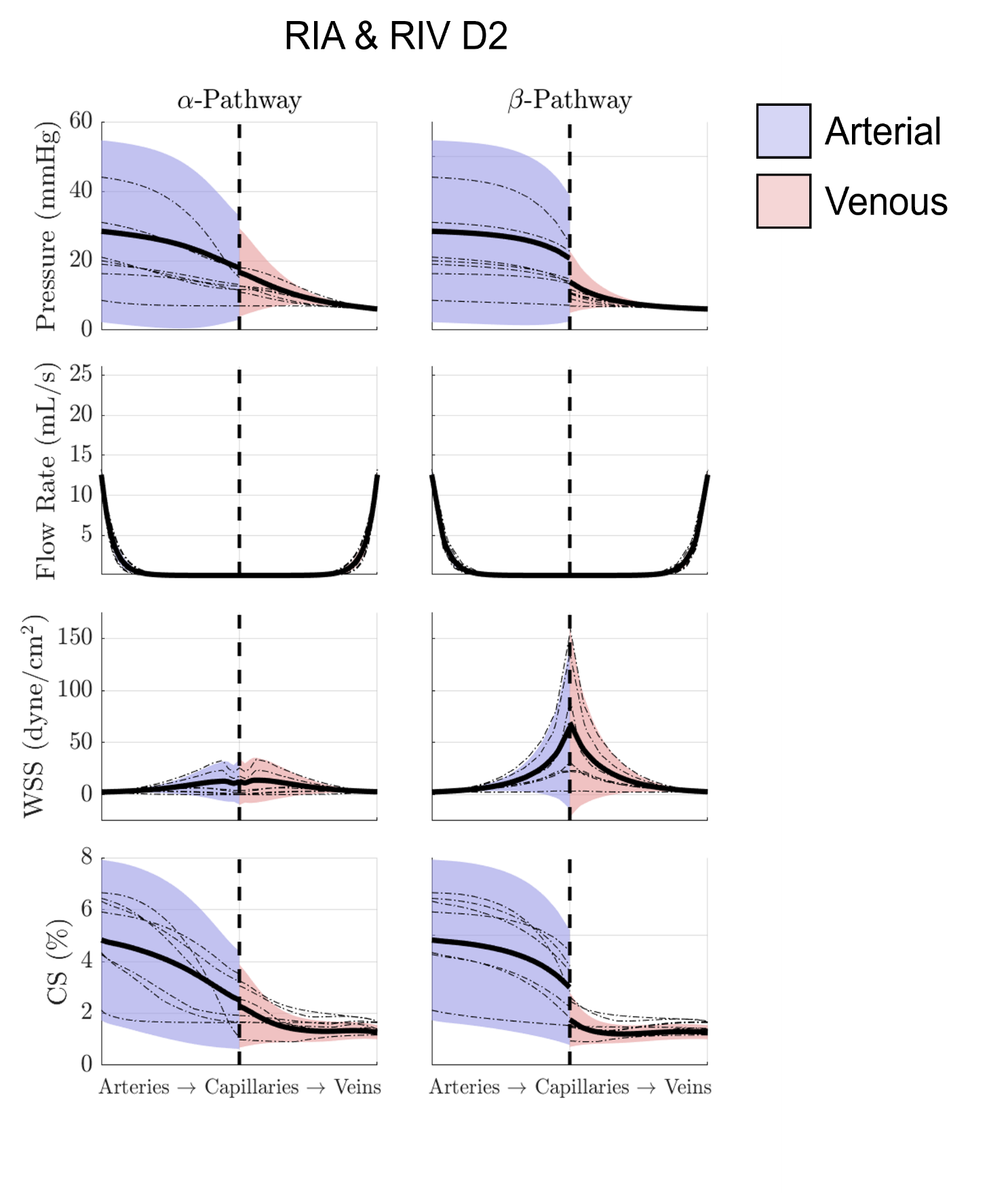


**Fig. S8:** Uncertainty in time-averaged pressure, flow, wall shear stress (WSS), and cyclic stretch (CS) using polynomial chaos expansions. Arterial predictions are provided in blue while venous uncertainty is shown in pink. See main text for more methodological details. RIA: right inferior artery; RIV: right inferior vein. Realizations from the sampling procedure are shown in dash-dotted lines


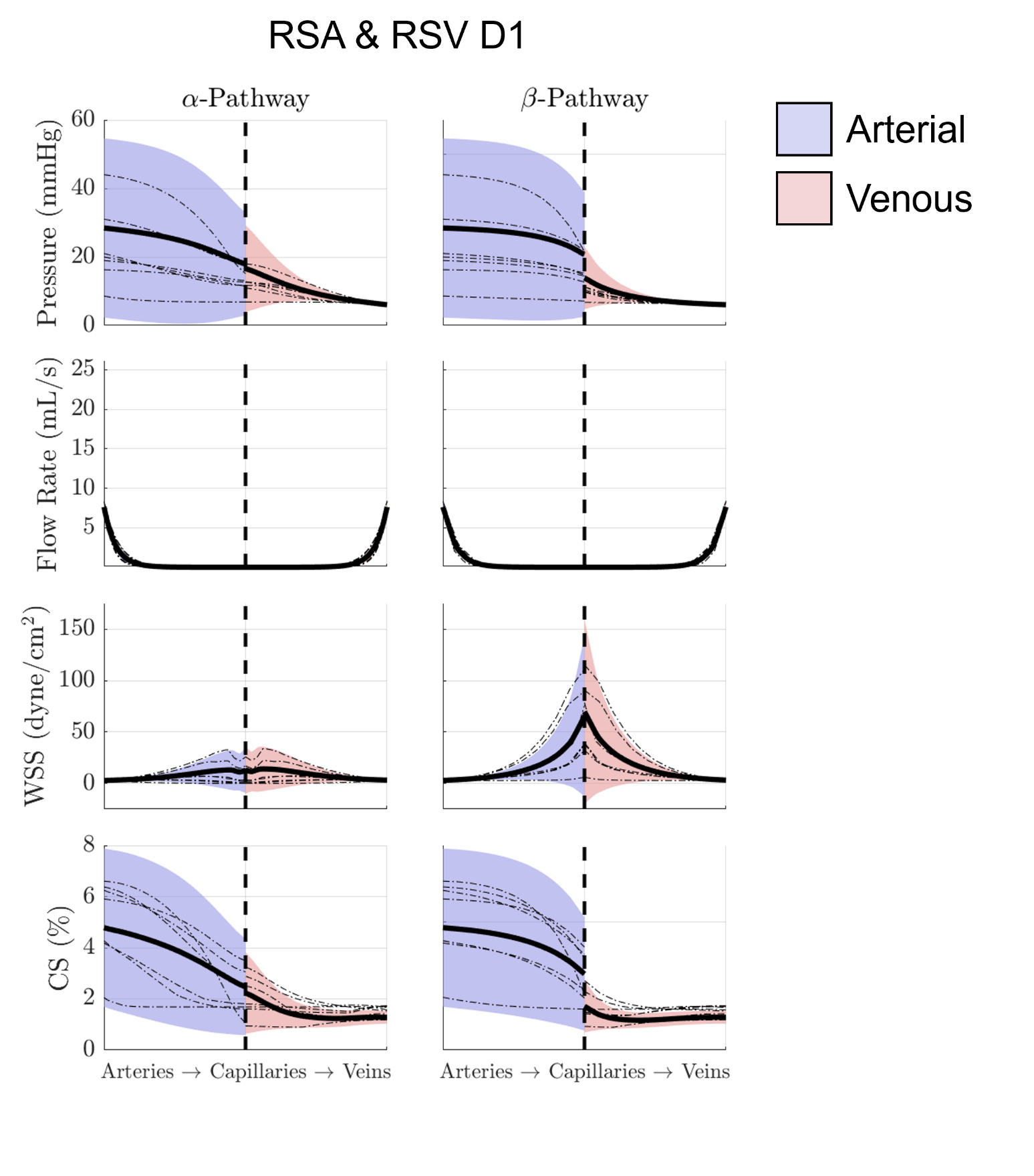


**Fig. S9:** Uncertainty in time-averaged pressure, flow, wall shear stress (WSS), and cyclic stretch (CS) using polynomial chaos expansions. Arterial predictions are provided in blue while venous uncertainty is shown in pink. See main text for more methodological details. RSA: right superior artery; RSV: right superior vein. Realizations from the sampling procedure are shown in dash-dotted lines


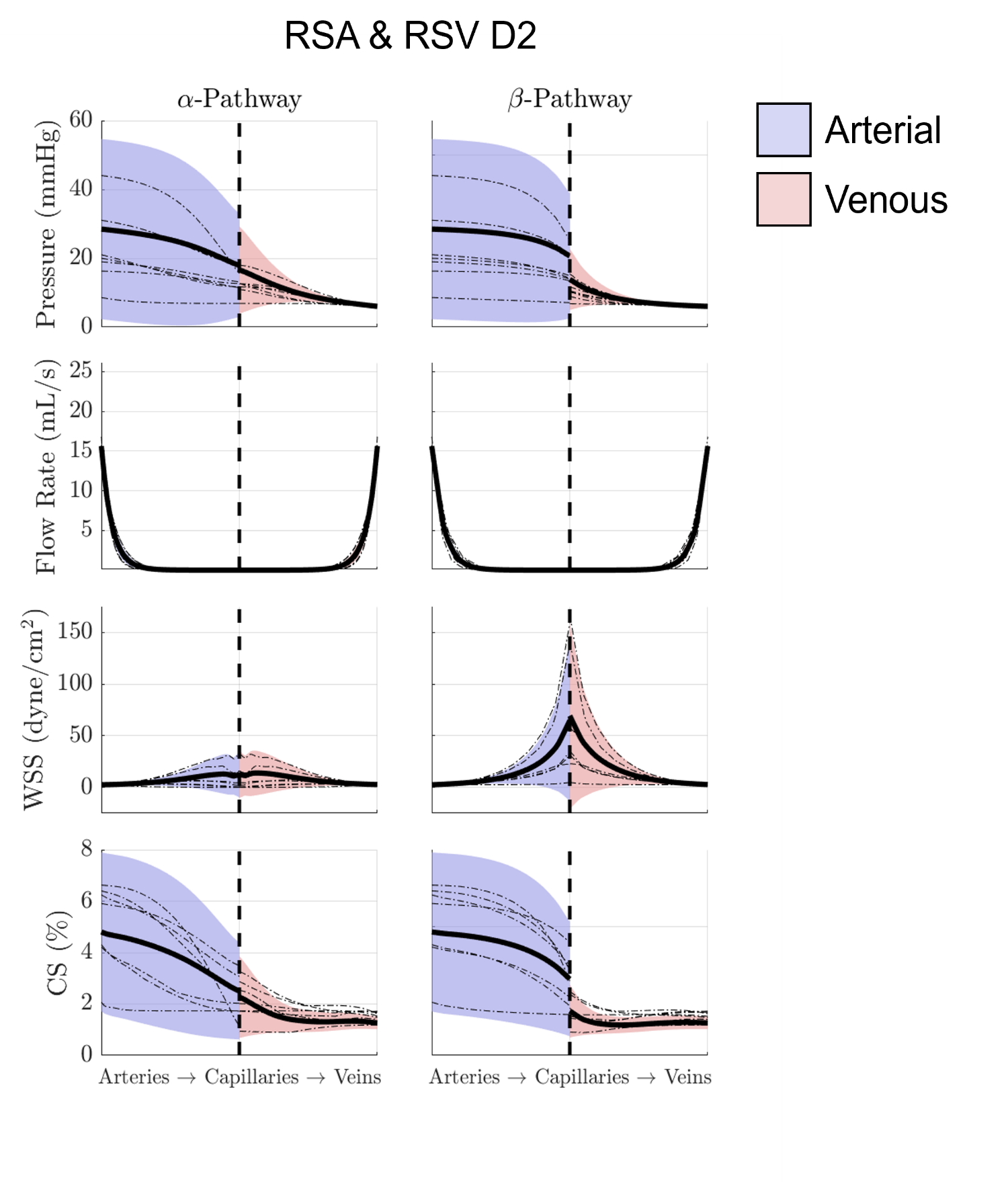


**Fig. S10:** Uncertainty in time-averaged pressure, flow, wall shear stress (WSS), and cyclic stretch (CS) using polynomial chaos expansions. Arterial predictions are provided in blue while venous uncertainty is shown in pink. See main text for more methodological details. RSA: right superior artery; RSV: right superior vein. Realizations from the sampling procedure are shown in dash-dotted lines


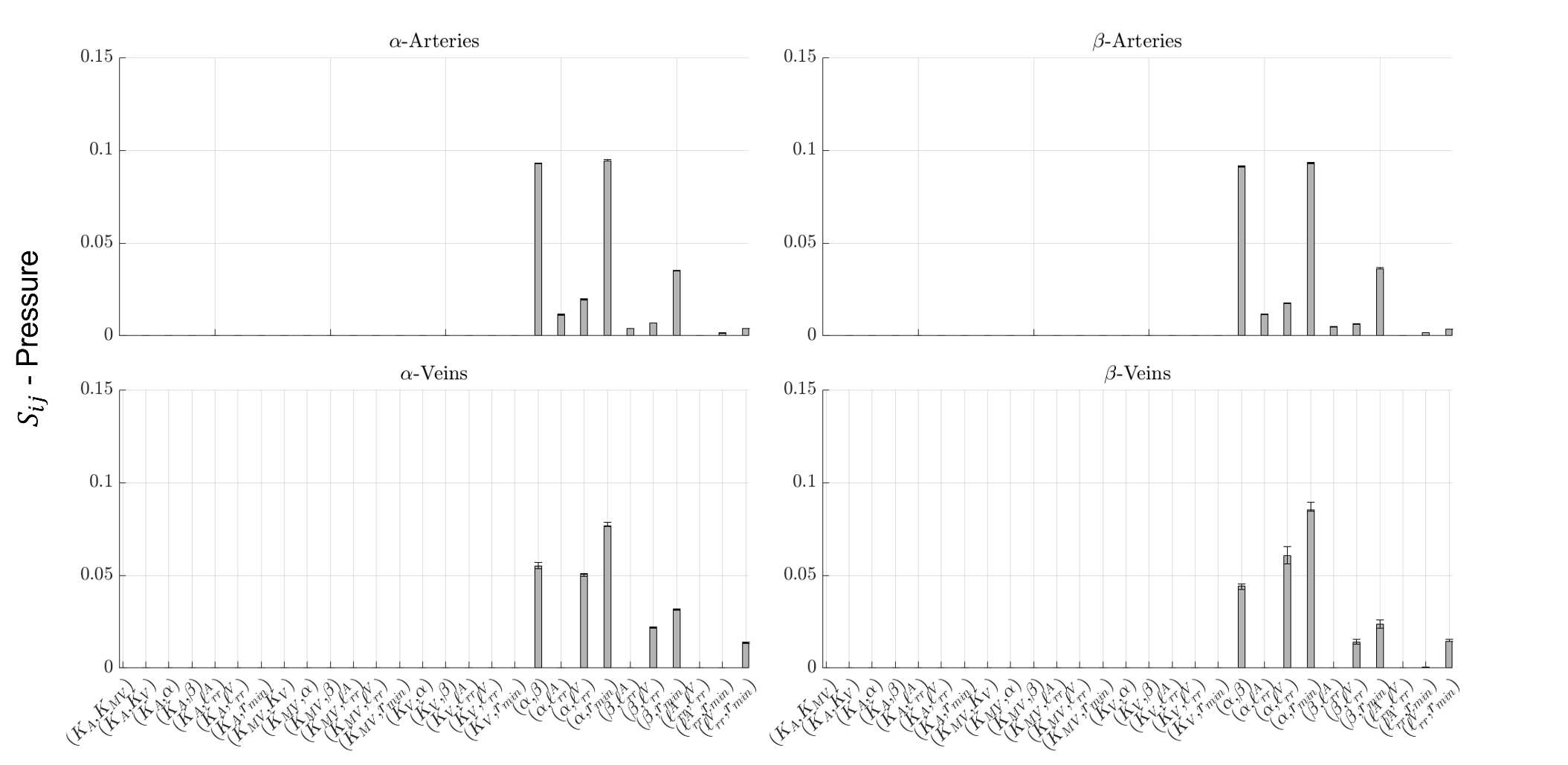


**Fig. S11:** Second-order Sobol’ indices, $S_{ij}$, for pressure in the $\alpha$ and $\beta$ pathways of the arterial or venous structured trees. Boxplots show the median value across all eight structured tree beds and the error bars show the range.


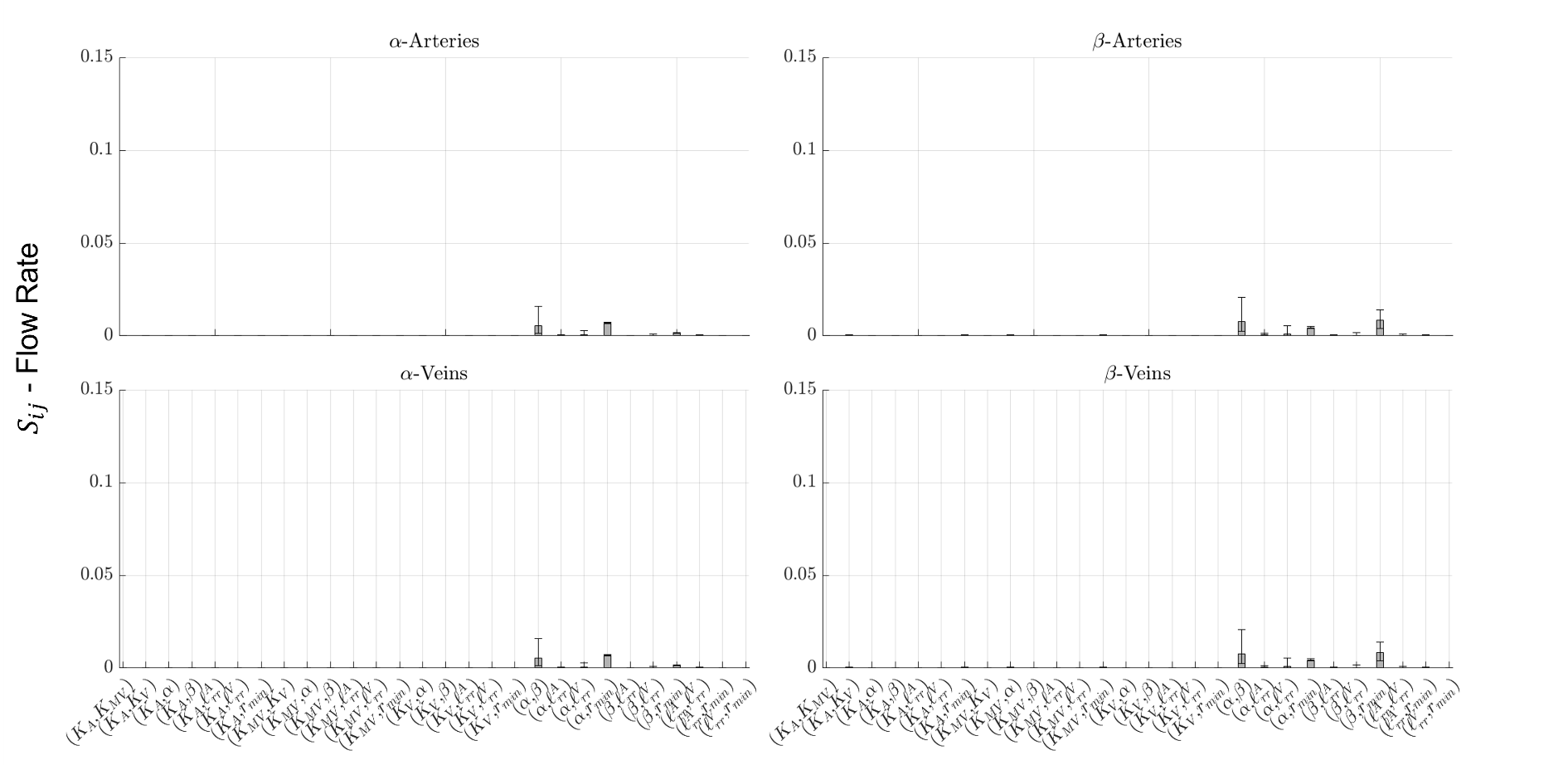


**Fig. S12:** Second-order Sobol’ indices, $S_{ij}$, for flow in the $\alpha$ and $\beta$ pathways of the arterial or venous structured trees. Boxplots show the median value across all eight structured tree beds and the error bars show the range.


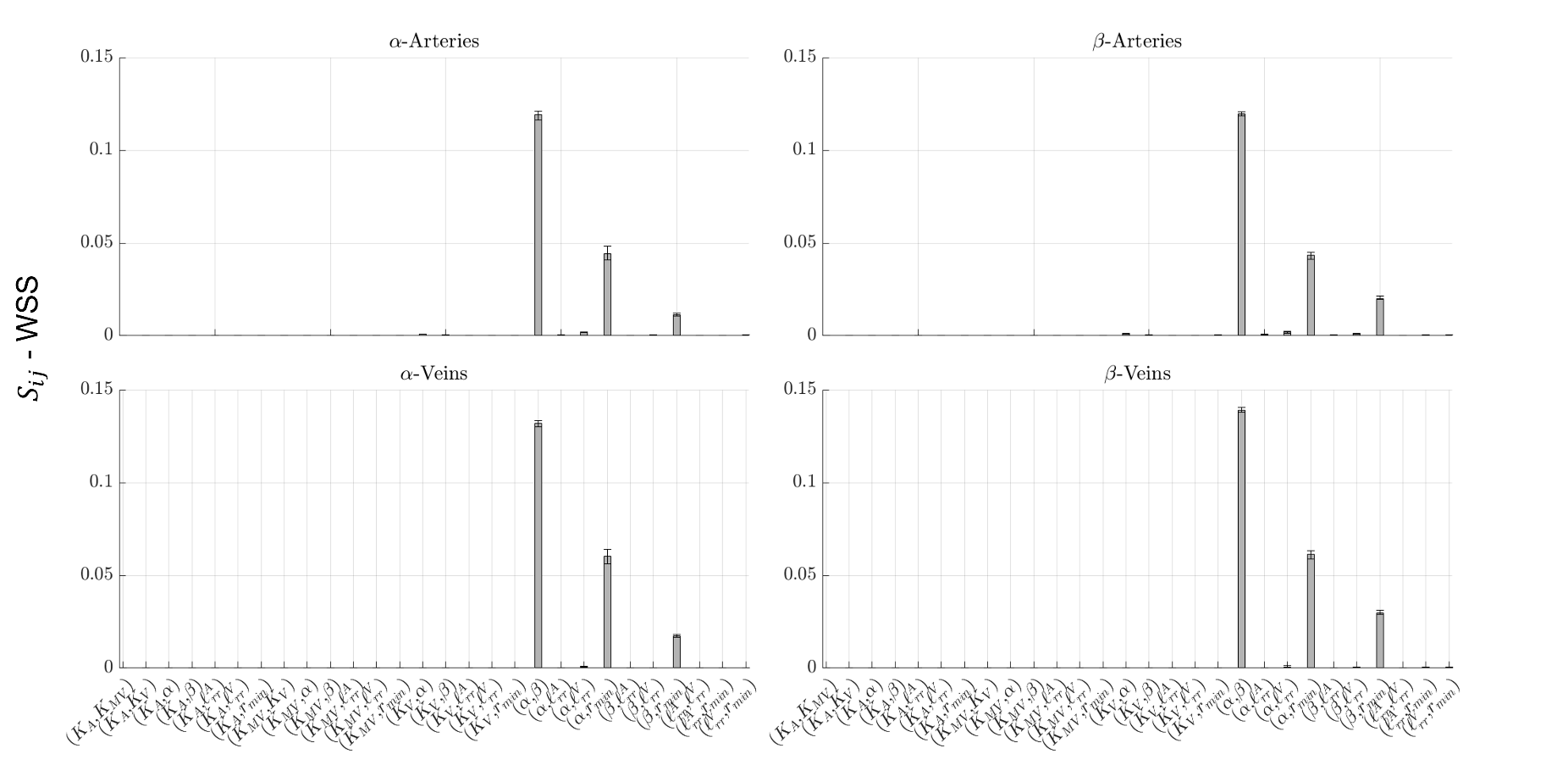


**Fig. S13:** Second-order Sobol’ indices, $S_{ij}$, for WSS in the $\alpha$ and $\beta$ pathways of the arterial or venous structured trees. Boxplots show the median value across all eight structured tree beds and the error bars show the range.


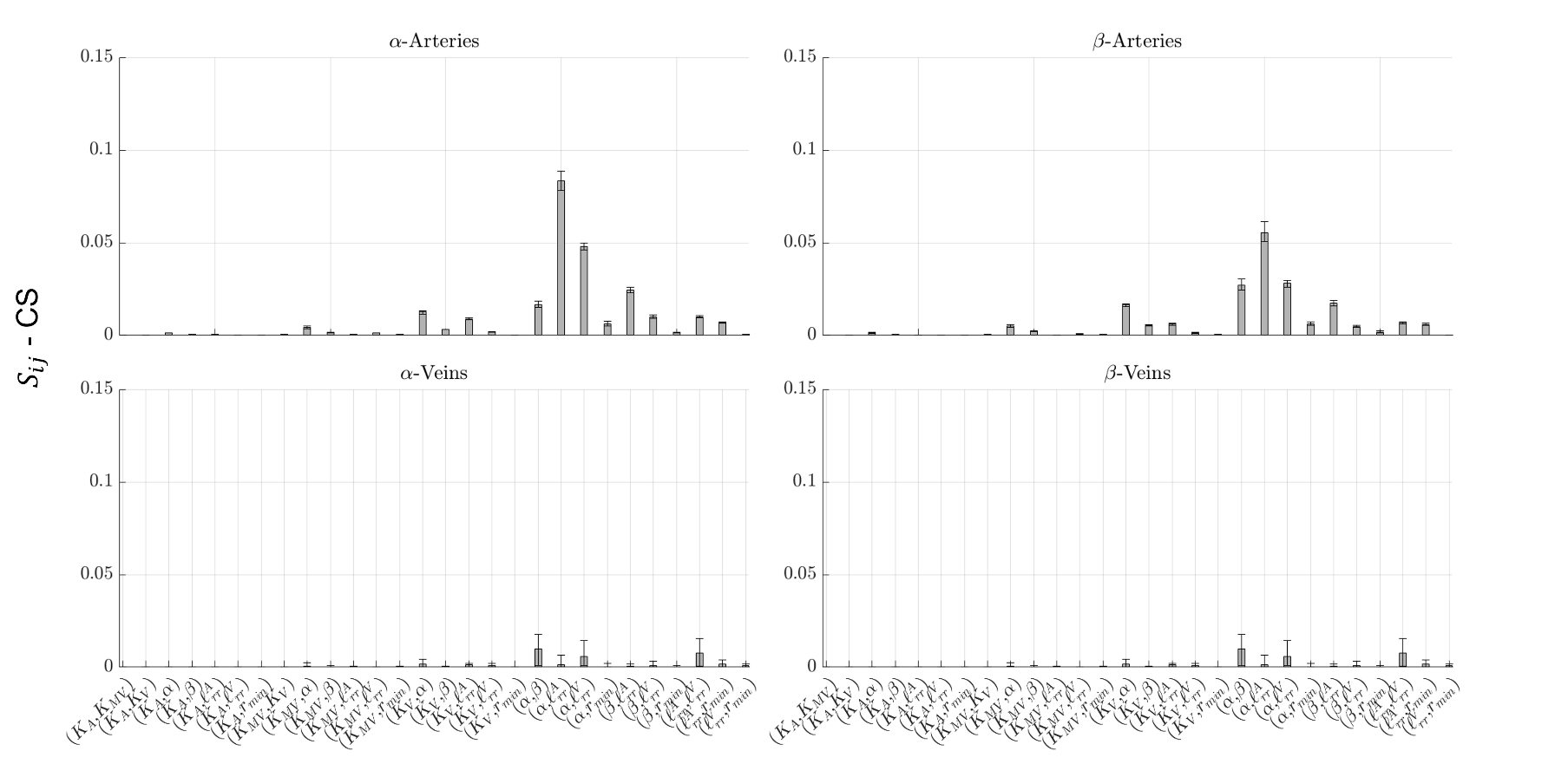


**Fig. S14:** Second-order Sobol’ indices, $S_{ij}$, for CS in the $\alpha$ and $\beta$ pathways of the arterial or venous structured trees. Boxplots show the median value across all eight structured tree beds and the error bars show the range.
